# Early inoculation of an endophyte alters the assembly of bacterial communities across rice plant growth stages

**DOI:** 10.1128/spectrum.04978-22

**Published:** 2023-09-01

**Authors:** Xing Wang, Shan-Wen He, Qing He, Zhi-Cheng Ju, Yi-Nan Ma, Zhe Wang, Jia-Cheng Han, Xiao-Xia Zhang

**Affiliations:** 1 Institute of Agricultural Resources and Regional Planning, Chinese Academy of Agricultural Sciences, Beijing, China; 2 Shanghai Academy of Landscape Architecture Science and Planning, Shanghai, China; 3 CAS Key Laboratory for Environmental Biotechnology, Research Center for Eco-Environmental Sciences, Chinese Academy of Sciences (CAS), Beijing, China; University of Texas at San Antonio, San Antonio, Texas, USA

**Keywords:** rice microbiome, priority effect, *Xanthomonas sacchari*, ecological process, endophytic bacteria

## Abstract

The core endophytes of plants are regarded as promising resources in future agroecosystems. How they affect the assembly of rice-related bacterial communities after early inoculation remains unclear. Here, we examined bacterial communities across 148 samples, including bulk and rhizosphere soils, sterilized roots, stems, and seeds at the seedling, tillering, booting, and maturity stages. Tissue cultured rice seedlings were inoculated with *Xathomonas sacchari* JR3-14, a core endophytic bacterium of rice seeds, before transplanting. The results revealed that α-diversity indices were significantly enhanced in the root and stem endosphere at the seedling stage. β-diversity was altered at most plant developmental stages, except for the root and stem at the booting stage. Network complexity consequently increased in the root and stem across rice growth stages, other than the stem endosphere at the booting stage. Four abundant beneficial bacterial taxa, *Bacillus*, *Azospira*, *Azospirillum*, and *Arthrobacter*, were co-enriched during the early growth stage. Infer Community Assembly Mechanisms by Phylogenetic-bin-based null model analysis revealed a higher relative contribution of drift and other eco-evolutionary processes mainly in root compartments across all growth stages, but the opposite pattern was observed in stem compartments.

**IMPORTANCE:**

Endophytic bacteria are regarded as promising environmentally friendly resources to promote plant growth and plant health. Some of microbes from the seed are able to be carried over to next generation, and contribute to the plant’s ability to adapt to new environments. However, the effects of early inoculation with core microbes on the assembly of the plant microbiome are still unclear. In our study, we demonstrate that early inoculation of the rice seed core endophytic bacterium *Xanthomonas sacchari* could alter community diversity, enhance complexity degree of network structure at most the growth stages, and enrich beneficial bacteria at the seedling stage of rice. We further analyzed the evolutionary processes caused by the early inoculation. Our results highlight the new possibilities for research and application of sustainable agriculture by considering the contribution of seed endophytes in crop production and breeding.

## INTRODUCTION

Endophytic bacteria can colonize in the internal tissues of plants. Some beneficial endophytes promote plant growth through direct or indirect effects. For example, they may directly enhance plant nutrient absorption, produce phytohormone to regulate plant growth, and adapt to environmental stress. Indirectly, they can protect plant by producing cell wall degrading enzymes, antibiotics, and activating plant defense mechanisms (1, 2). They can thrive in different compartments of plants including roots, stems, leaves, flowers, fruits, and seeds (3). Many previous studies have concentrated on the rhizosphere microbiome due to its importance to plant growth and health (4, 5); however, our understanding of the seed microbiome has lagged far behind that of root-associated microbes (6 – 10).

Microbes from the seeds may affect germination, seedling development, and plant growth, and the microbes may be carried over to the next generation (6, 7, 11, 12). Compared with bacteria originating from the outside environment, seed endophytes are more likely to reside within plant tissues and rapidly colonize the emerging plants due to lower competition for space and the greater available nutrients in the host plant (13 – 15). The seed endophytes in turn contribute to the plant’s ability to adapt to a new environment, for example, Shao et al. reported that seed-borne bacteria could be used as a functional compensation reservoir to promote the acquisition of nutrients in nutrient-deficient soils (16).

Core microbes are regarded as promising resources to increase the resource efficiency and stress resistance of future agroecosystems. In view of the importance of plant microbial priority effect, the contribution of core microorganisms to the management of microbial assembly in seeds and seedlings should be the focus of research (17). According to Hacquard et al., compared with the adult plant, core microbial species were more likely to establish in the root or rhizosphere during the seed and seedling stages (18). Many studies have shown that rice seeds typically harbor “*Xanthomonas*” as core members (19 – 22). Our group also found that *Xanthomonas* was the common dominant genus in five different genotypes of rice seeds using high-throughput sequencing technology (23). Although members of the genus *Xanthomonas* are mostly known for their pathogenic behavior (24, 25), many new non-pathogenic strains have been observed during the last decade (26 – 31), including *Xanthomonas sacchari*. The type III secretion system (T3SS) and type VI secretion system (T6SS) have been identified to influence host specificity and bacterial pathogenicity in most *Xanthomonas* spp. (32, 33); however, *X. sacchari* possesses atypical T3SS and T6SS (25). Previous studies have shown that most *X. sacchari* strains were isolated or detected from rice seeds (34 – 36). We investigated the high similarity of 16S rRNA gene with *X. sacchari* in NCBI database showed that 75% strains were derived from rice plants, and half of them were derived from rice seeds (Table S1). *X. sacchari* R1 has been used as a biocontrol agent due to its antagonistic ability against *Burkholderia glumae* (29). We previously conducted a large-scale high-throughput sequencing analysis on microbial communities from five different rice varieties inhabiting five microenvironments (bulk soil, rhizosphere soil, roots, stems, and seeds) from four geographical locations. Combined with comparative genomic analysis, *Xanthomonas* was identified as a vertically transmitted core endophytic bacterium in rice seeds (37). Therefore, *X. sacchari* is regarded as an ideal organism to study how plant microbiome assembly is affected by early inoculation.

The current concept of the “holobiont” supposes that plants and their associated microbiomes form an assemblage of species, whose ecology and evolution are considered to be intertwined (38, 39). Community diversity and dynamics in plant-associated microbiota are controlled by four fundamental eco-evolutionary processes: selection, dispersal, speciation or diversification, and ecological drift (40 – 42). Quantifying the relative importance of eco-evolutionary processes plays non-negligible roles for unraveling the drivers controlling community assembly (42 – 44). Several recent investigations have provided quantitative insight into how ecological processes control plant-associated microbial communities residing in different microhabitats (45 – 47). For instance, application of iCAMP (Infer Community Assembly Mechanisms by Phylogenetic-bin-based null model) to the microbial communities in response to drought stress revealed the increase of the stochastic process of drift in the aboveground compartments of plants, and the reduction of stochastic processes of dispersal limitation and drift in belowground compartments (48). However, it is unclear how early inoculation with endophytic bacteria during the early stages affects the ecological process of controlling microbial community structure.

In this study, we aimed to clarify the effects of early inoculation of rice seeds with the endophyte *X. sacchari* on the bacterial community structure and ecological processes which control the assembly of endosphere and rhizosphere microbiomes. Here, we primarily focused on the following areas: first, comprehensive analysis of how the early inoculation impacts the microbial community structure at compartments of rice plants during different growth stages; second, which ecological processes (selection, dispersal, diversification, drift, and others) determined the composition of microbial communities under early inoculation conditions. The results will have relevant implications for the concepts of plant microbiome ecology and the holobiont, as well as providing new possibilities for research and application of seed endophytes in future agriculture.

## RESULTS

### Early inoculation changes the microbial composition

After quality filtering, the remaining 12,956 unique sequences and 8,750,523 reads were obtained among 148 samples. The low reads were discarded, and rice plant mitochondria, chloroplast, archaea, and unassigned (phylum level) were filtered, resulting in a total of 6,687 bacterial zero-radius operational taxonomic units (ZOTUs).

**Fig 1:**
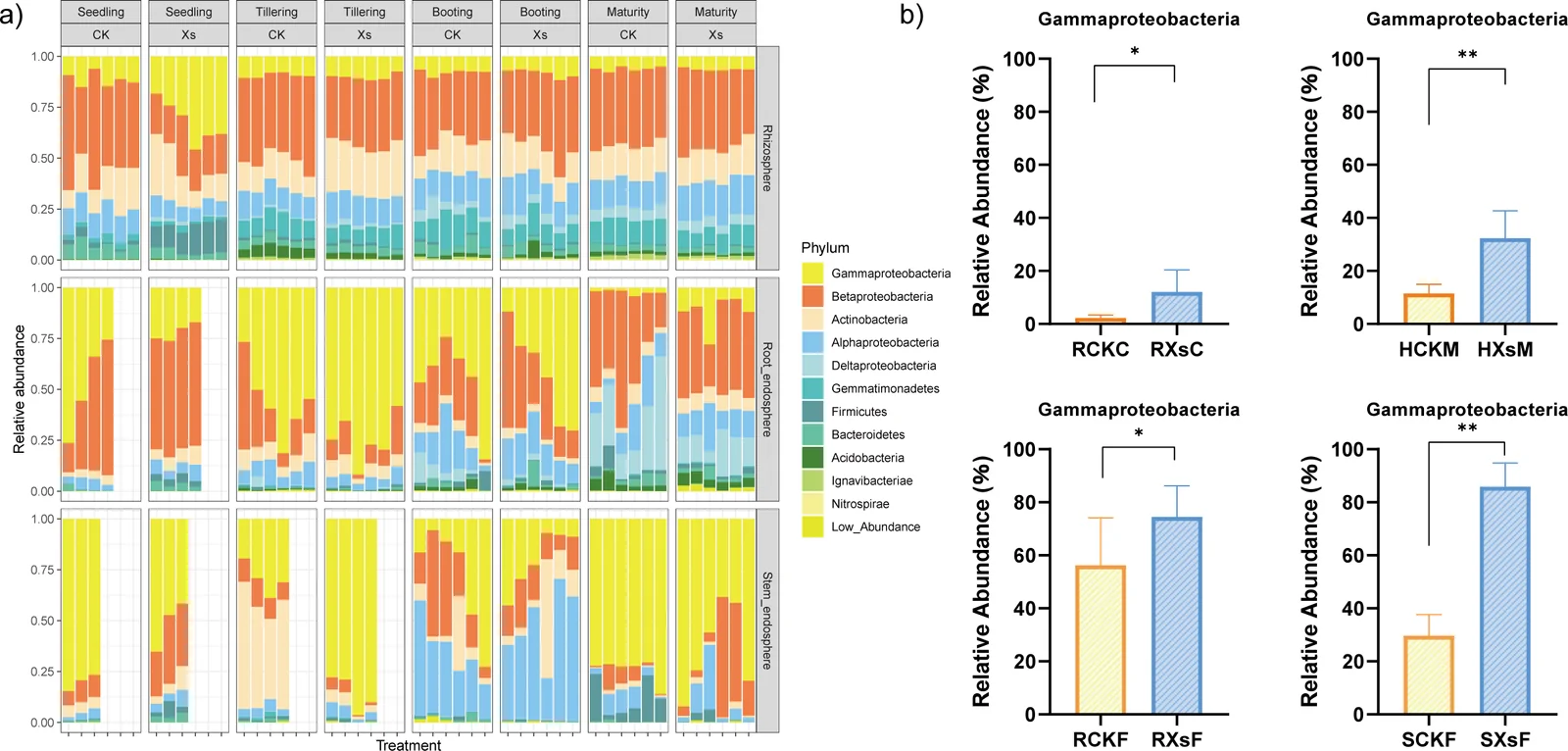
Taxonomic composition of the bacterial communities. (**a**) The relative abundances of the most abundant (sub)phylum level in each compartment between Xs and CK. (**b**) Comparative analysis of relative abundance of Gammaproteobacteria in Xs and CK (paired *t*-test). “RCKC/ RXsC,” root endophytic microbes CK or Xs at maturity stage; “HCKM/ HXsM,” rhizosphere microbes CK or Xs at seedling stage; “RCKF/RXsF,” root endophytic microbes CK or Xs at tillering stage; “SCKF/SXsF,” stem endophytic microbes CK or Xs at tillering stage. The statistical analyses were performed using a two-sided *t*-test. *P*-values are indicated by *. * represents *P* < 0.05, ** represents *P* < 0.01.

Comprehensive exploration of community composition was conducted first among the developmental stages. We found that rice microbiomes in CK and Xs consisted mostly of Gammaproteobacteria, Betaproteobacteria, Actinobacteria, Alphaproteobacteria, and Deltaproteobacteria, accounting for >90% of total relative abundance (Fig. 1a), with Gammaproteobacteria, Actinobacteria, and Betaproteobacteria dominating the seed-associated bacterial assemblages (Fig. S1). Except for the seedling stage, the composition of microbiomes at the (sub)phylum level in the rhizosphere and bulk soil were evenly distributed between treatment (Xs) and control (CK). Therefore, we paid more attention to the relative abundance of (sub)phylum species in the endophytic compartments after early inoculation compared to CK. The results showed that relative abundance of Gammaproteobacteria were significantly increased in rhizosphere at seedling stage (*P* < 0.01), and in the root at tillering stage (*P* < 0.05) and maturity stage (*P* < 0.05) (Fig. 1b). Notably, Gammaproteobacteria were more abundant in Xs (77.86%–96.44%) than in CK (19.49%–38.87%) in the stem at the tillering stage after early inoculation (*P* < 0.01). These results indicated that early inoculation of *X. sacchari* in the roots of rice leads to the dominance of Gammaproteobacteria as the prevalent microbial community in the rhizosphere during seedling stage. This phenomenon was also observed in the roots and stems during the tillering stage.

### Early inoculation affects the diversity of rice microbiome

**Fig 2:**
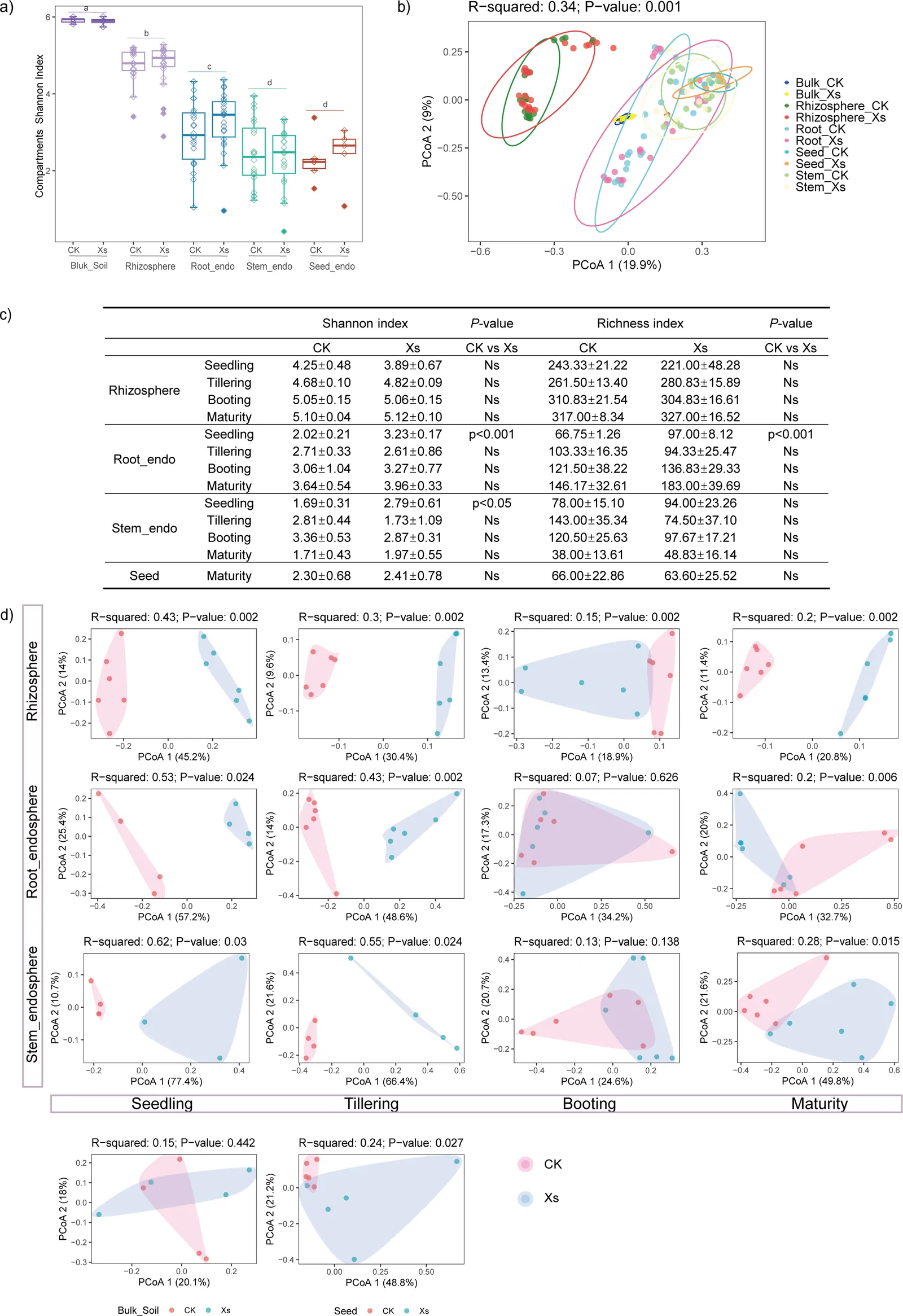
Taxonomic α- and β-diversity estimates. (**a**) Shannon indices of bacterial communities in bulk soil, rhizosphere, root endosphere, stem endosphere, and seed endosphere. Horizontal lines within boxes denote medians. Tops and bottoms of boxes denote the 75th and 25th percentiles, respectively. Upper and lower whiskers extend to data no more than 1.5 times the interquartile range from the upper edge and lower edge of the box, respectively. Different letters indicate significant differences among compartments (*P* < 0.05), based on Kruskal-Wallis one-way test. (**b**) PCoA plot depicting the β-diversity patterns of bacterial communities across different compartments based on Bray-Curtis dissimilarity. (**c**) α-Diversity CK in each compartment at the seedling stage, tillering stage, booting stage, and maturity stage compared with Xs based on the Wilcoxon rank sum test. Shannon and richness index represent mean ± SD according to group, “Ns” represents not significantly different. (**d**) PCoAs of microbial community composition in individual compartments at each development stage between Xs and CK based on the Bray-Curtis dissimilarity. Significant difference between treatments with and without *Xanthomonas* inoculation based on permutational multivariate analysis of variance (PERMANOVA) and adonis function.

To evaluate the diversity of the rice-associated microbiomes, α-diversity (Shannon and richness index) was calculated at the ZOTU level between Xs and CK. We comprehensively analyzed α-diversity of rice-related microbial communities in each of the different compartments (bulk soil, rhizosphere, and endosphere of roots, stems, and seeds) as a whole. The results suggested that the microbial α-diversity indices of various plant compartments decreased significantly in the order of bulk soil, rhizosphere, root, and stem regardless of early inoculation treatment (*P* < 0.001) (Fig. 2a). At the same time, principal coordinate analysis (PCoA) was conducted to visualize the β-diversity based on the Bray-Curtis dissimilarity (Fig. 2b). The results showed that the microbial community was divided into different clusters according to the host compartments (*P* < 0.001), indicating that early inoculation could not affect the spatial compartmentalization of bacterial microbial communities.

Next, we sought to determine the effects of early inoculation of an endophyte on driving microbiome assemblages at each developmental stage (seedling stage, tillering stage, booting stage, and maturity stage) during whole growth stages. Therefore, we compared and calculated α-diversity and β-diversity of microbial communities between CK and Xs in individual compartments from seedling stage to maturity stage, respectively. We found that Shannon indices of Xs were significantly higher than those of CK in the root (*P* < 0.001) and stem (*P* < 0.05) endospheres at the seedling stage, while there was no significant difference in other developmental stages (Fig. 2b). Compared with CK, no changes in α diversity were observed in all developmental stages of rhizosphere, and it was not found in endophytic rice seeds either. This indicates that inoculating core endophytic bacteria in seeds only increases the α diversity of bacteria in the initial growth stage of endophytic roots and stems, but it does not continue in the subsequent growth stages (Fig. 2c).

The early inoculation had non-significant influence on the bulk soil bacterial community, which indicated that the rice-related microbial communities were more susceptible to early inoculation than bulk soil (PERMANOVA: *P* > 0.05) (Fig. 2d). The results of PCoA of the full data set suggested that assembly of the rhizosphere microbial community mainly occurred at the seedling (*R* ^2^ = 0.43, *P* < 0.002) and tillering (*R* ^2^ = 0.30, *P* < 0.002) stages, then at the maturity stage (*R* ^2^ = 0.20, *P* < 0.002). For microbial community assembly of the root endosphere, the highest explanation was at the seedling stage (*R* ^2^ = 0.53, *P* < 0.024), followed by the tillering stage (*R* ^2^ = 0.43, *P* < 0.002) and maturity stage (*R* ^2^ = 0.20, *P* < 0.006), and a similar pattern was also observed in the stem endosphere from seedling stage (*R* ^2^ = 0.62, *P* < 0.030), tillering stage (*R* ^2^ = 0.55, *P* < 0.024) to maturity stage (*R* ^2^ = 0.28, *P* < 0.015) (Fig. 2d). However, the results in root and stem at the booting stage did not follow the pattern described above. Most notably, early inoculation had significant influence on the bacterial community in the seed endosphere but only accounted for slight variation (*R* ^2^ = 0.18, *P* < 0.027) (Fig. 2d). Together, these results indicated that early inoculation altered the bacterial community β-diversity in each compartment over the rice plant growth stages except for the root and stem at the booting stage.

### Early inoculation increased the microbial co-occurrence network complexity and positive interconnections

**Fig 3:**
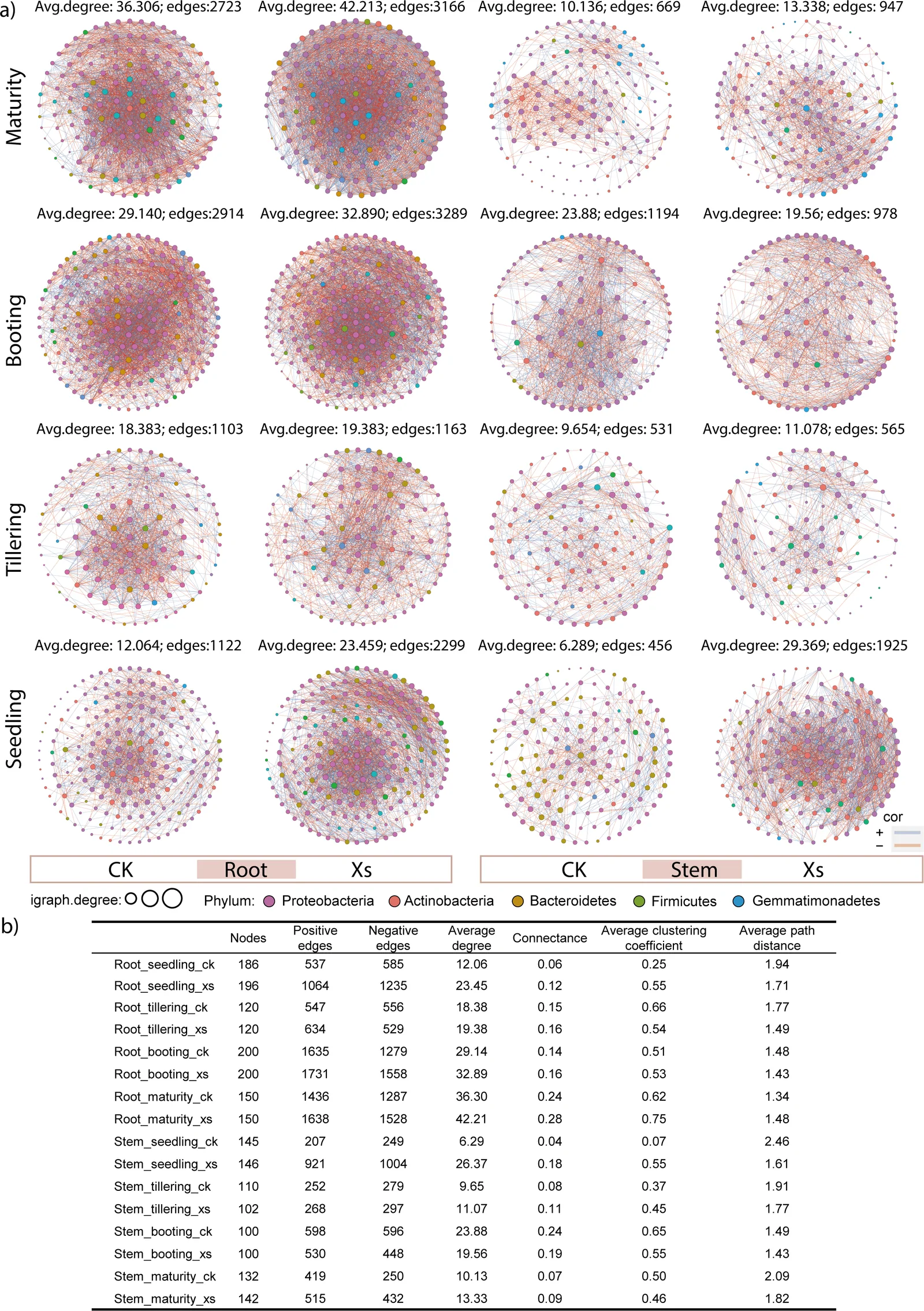
Effect of early inoculation on network complexity and positive related connections. (**a**) Bacterial co-occurrence networks in root and stem endophytic compartments (90 samples). (**b**) Bacterial co-occurrence network characteristics in each compartment niche. The size of each node represents the size of average degree of the microbe. Link color indicates the type of correlation: blue, positive correlation; red, negative correlation.

To further characterize the effects of early inoculation on plant microbiomes, particularly for endophytes (seed, stem, and root), we assessed the co-occurrence patterns of bacterial communities at each developmental stage. A higher average degree represents a higher network complexity (49). Our results suggested that the complexity of the network constructed by early inoculation was higher, with exception of the stem compartment at the booting stage (Fig. 3a and b). Specifically, the network complexity of early inoculation was higher than that of CK in root at the seedling stage (with an average degree of 12.06 in CK and 23.45 in Xs), tillering stage (18.38 in CK and 19.38 in Xs), booting stage (29.14 in CK and 32.89 in Xs), and maturity stage (36.30 in CK and 42.21 in Xs). Correspondingly, apart from stems at booting stage, the complexity of bacterial network was also higher than that of CK at seedling stage (6.29 in CK and 26.37 in Xs), tillering stage (9.65 in CK and 11.07 in Xs), and maturity stage (10.13 in CK and 13.33 in Xs). In addition, the network complexity constructed by Xs within the seed endosphere was higher than that of CK (Fig. S2).

Furthermore, analysis of topological features showed that positive interconnections in the early inoculation were greater than that of CK except for in the stem at the booting stage according to positive edges index. These results indicated that more complex network structure and far more positive interconnections were observed after early inoculation with exception of stems data at the booting stage.

### Early inoculation enriched more microbes at the seedling stage

**Fig 4:**
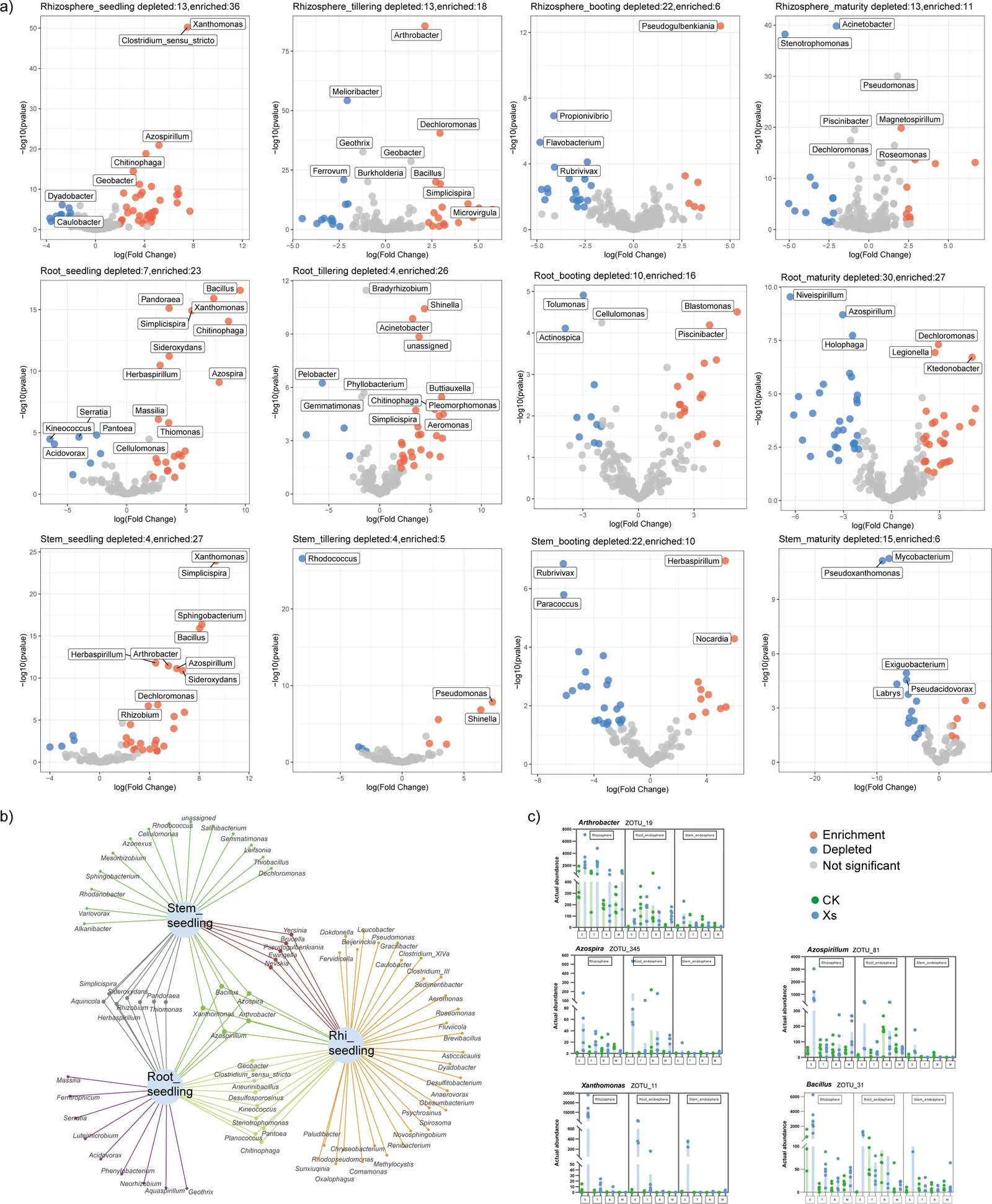
Enrichment of genera inhabiting different plant microhabitats. (**a**) The volcano plot illustrates the enrichment and depletion of microbial communities in each compartment of Xs compared to CK at each growth stage using the deseq2 package. Genera were considered enriched if they showed a log2 fold change greater than two and an adjusted *P*-value <0.05. Each point represents a genus. Each red point represents enriched genus, and blue points represent depleted genera. (**b**) Venn plot illustrating the five genera that were significantly co-enriched at the seedling stage compared with CK in root endosphere, stem endosphere, and rhizosphere. (**c**) The distribution pattern of actual abundance of the four co-enriched genera in each compartment at the developmental stages. The letter S in x-axis represents seedling stage; T, tillering stage; B, booting stage; M, maturity stage.

Compared to CK, the number of genera enriched in root endosphere, stem endosphere, and rhizosphere during seedling and tillering stages was higher in Xs than the number of depleted genera (Fig. 4a; Table S2). The volcano plot only shows partial labels of the genus, and complete information can be found in Table S2. For example, 36 genera were enriched and 13 genera were depleted in the rhizosphere at the seedling stage compared with CK. These results suggest that early inoculation of a single endophytic bacterium in rice roots resulted in the recruitment of more genera in each plant compartment of the initial growth stage. Furthermore, we analyzed the co-enriched taxa among different rice compartments during the same growth stage, which may play important roles in plant growth. We found co-enriched genera in the rhizosphere, root endosphere, and stem endosphere during the seedling stage.

Four genera, *Bacillus*, *Azospira*, *Azospirillum*, and *Arthrobacter*, were co-enriched with *Xanthomonas* in rhizosphere, root endosphere and stem endosphere (Fig. 4b). Overall, the actual abundance of the co-enriched genus in Xs and CK showed a decreasing trend in detection rates from the rhizosphere, root endosphere, to stem endosphere in the four developmental stages (Fig. 4c). Notably, we found that the actual abundance of *Xanthomonas* in the Xs was significantly higher than that in CK after inoculation of *X. sacchari* JR3-14 at the seedling stage in rhizosphere (*P*_adj_ < 0.001), in roots (*P*_adj_ < 0.001), and in stems (*P*_adj_ <0.001). This was consistent with our hypothesis that the actual abundance of *Xanthomonas* would rise after early inoculation. At the same time, the 16S rRNA gene sequence extracted from whole genome of *X. sacchari* JR3-14 was identical to ZOTU_11 (*Xanthomonas*). In comparison with CK, *Bacillus*, *Azospira*, *Azospirillum*, and *Arthrobacter* were significantly co-enriched at the seedling stage in rhizosphere (*P*_adj_ < 0.001, *P*_adj_ < 0.001, *P*_adj_ < 0.001, *P*_adj_ < 0.001, respectively), root (*P*_adj_ < 0.001, *P*_adj_ < 0.001, *P*_adj_ < 0.01, *P*_adj_ < 0.001, respectively), and stem (*P*_adj_ < 0.001, *P* < 0.05, *P*_adj_ < 0.01, *P*_adj_ < 0.001, respectively) (Fig. 4c). Moreover, we performed functional annotation of prokaryotic taxa (FAPROTAX) analysis to predict functional profiles of the four co-enriched microbiotas. We found that some functions were more abundant in Xs such as aerobic_chemoheterotrophy, nitrate_respiration, nitrate_reduction, and chemoheterotrophy compared to CK (Fig. S3). These results suggest that inoculating a core endophytic bacterial strain in the early stages can recruit more beneficial microbiota to the rhizosphere, roots, and stems of rice.

### Microbial community assembly processes

**Fig 5:**
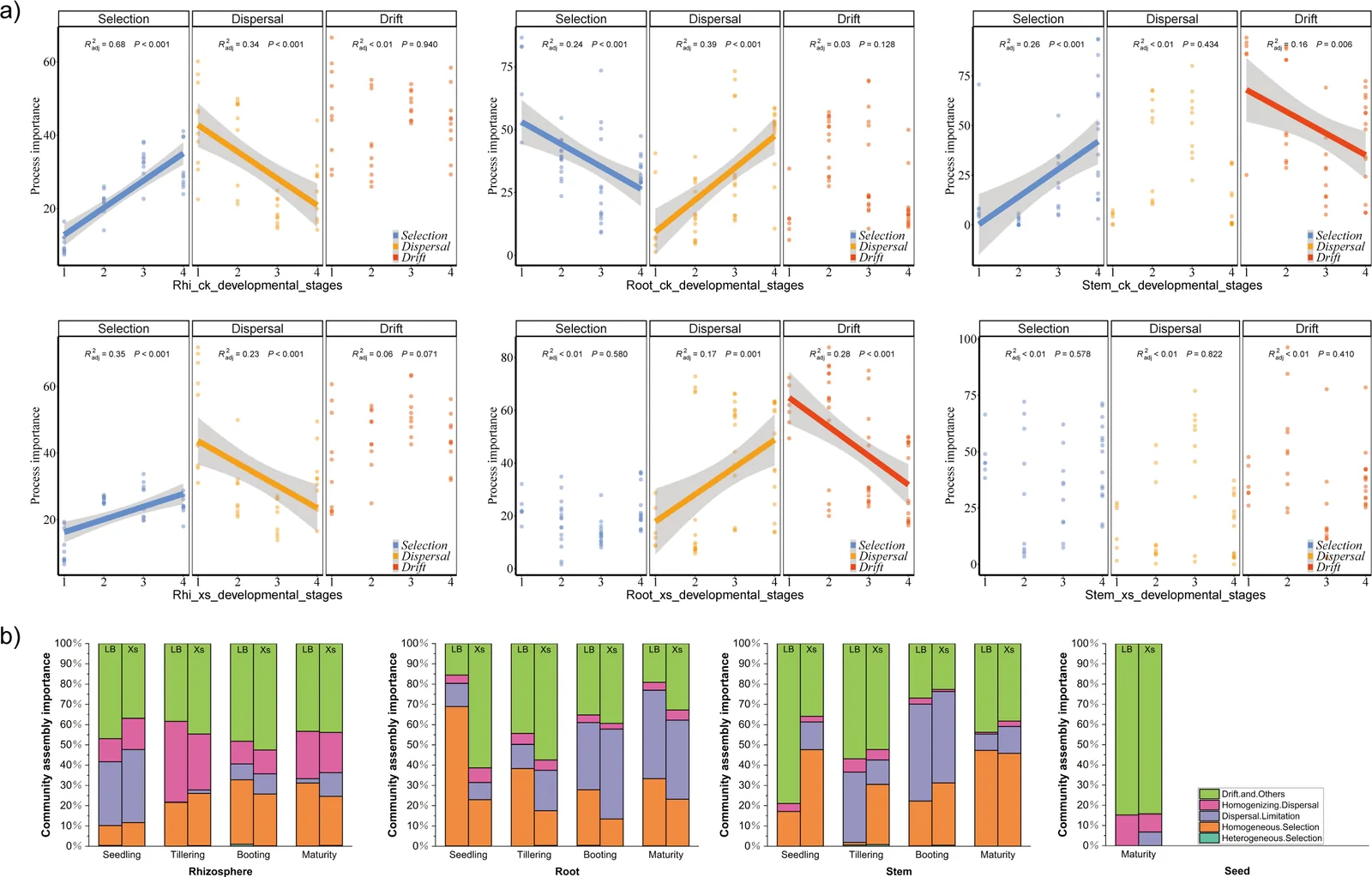
The microbial community assembly processes. (**a**) The percentage of turnover in the rhizosphere, root, and stem microbial communities, governed by “selection,” “dispersal,” and “drift” processes. The position along the y-axis represents the process importance (“selection” is a combination of homogeneous selection and heterogeneous selection; “dispersal” is a combination of homogenizing dispersal and dispersal limitation; “drift” represents drift and others), and the x-axis represents the growth stages: 1, seedling stage; 2, tillering stage; 3, booting stage; 4, maturity stage. (**b**) The community assembly importance in the rhizosphere, root, stem, and seed microbial communities governed by homogeneous selection, heterogeneous selection, dispersal limitation, homogenizing dispersal, and drift processes.

In order to reveal the ecological drivers of microbial community assembly of stem, root, and rhizosphere after early inoculation, the temporal dynamics of community assembly processes were quantitatively inferred by iCAMP. In the rhizosphere, the magnitude changes of dispersal (dispersal limitation and homogenizing dispersal), selection (heterogeneous selection and homogeneous selection), and drift (drift and others) processes showed similar trends in Xs and CK over the four growth stages (Fig. 5a). Through the column chart, we found that drift and other processes exerted a greater influence on the root and stem bacterial communities at all growth stages, respectively. In root compartments, the relative importance of “drift and others” process was higher in Xs (61.3%, 57.4%, 39.3%, 32.7%) than in CK (15.5%, 44.3%, 35.1%, 19.1%) at the seedling, tillering, booting, and maturity stages, respectively. Conversely, in the stem compartment, the relative contribution of “drift and others” ecological process was lower in Xs (35.9%, 52.2%, 22.6%, 38.2%) than in CK (78.9%, 56.8%, 26.8%, 43.7%) among the four developmental stages (Fig. 5b). Overall, iCAMP analysis revealed that both the root and stem communities were governed by the drift and others’ assembly process. Specifically, early endophyte inoculation decreased “drift and others” in the stem, but enhanced it in the root.

## DISCUSSION

The endophytic bacteria in seeds have the advantage of early colonization in roots and the rhizosphere, implying that early inoculation of endophytic bacteria may affect the assembly of the later plant microbiome. However, the ecological processes involved are still largely unclear. The current study used a core seed endophytic bacterium, *X. sacchari* JR3-14, as inoculant to explore the effects of early inoculation on the plant microbiome as well as the ecological processes of endosphere and rhizosphere microbiomes assembly.

**Fig 6:**
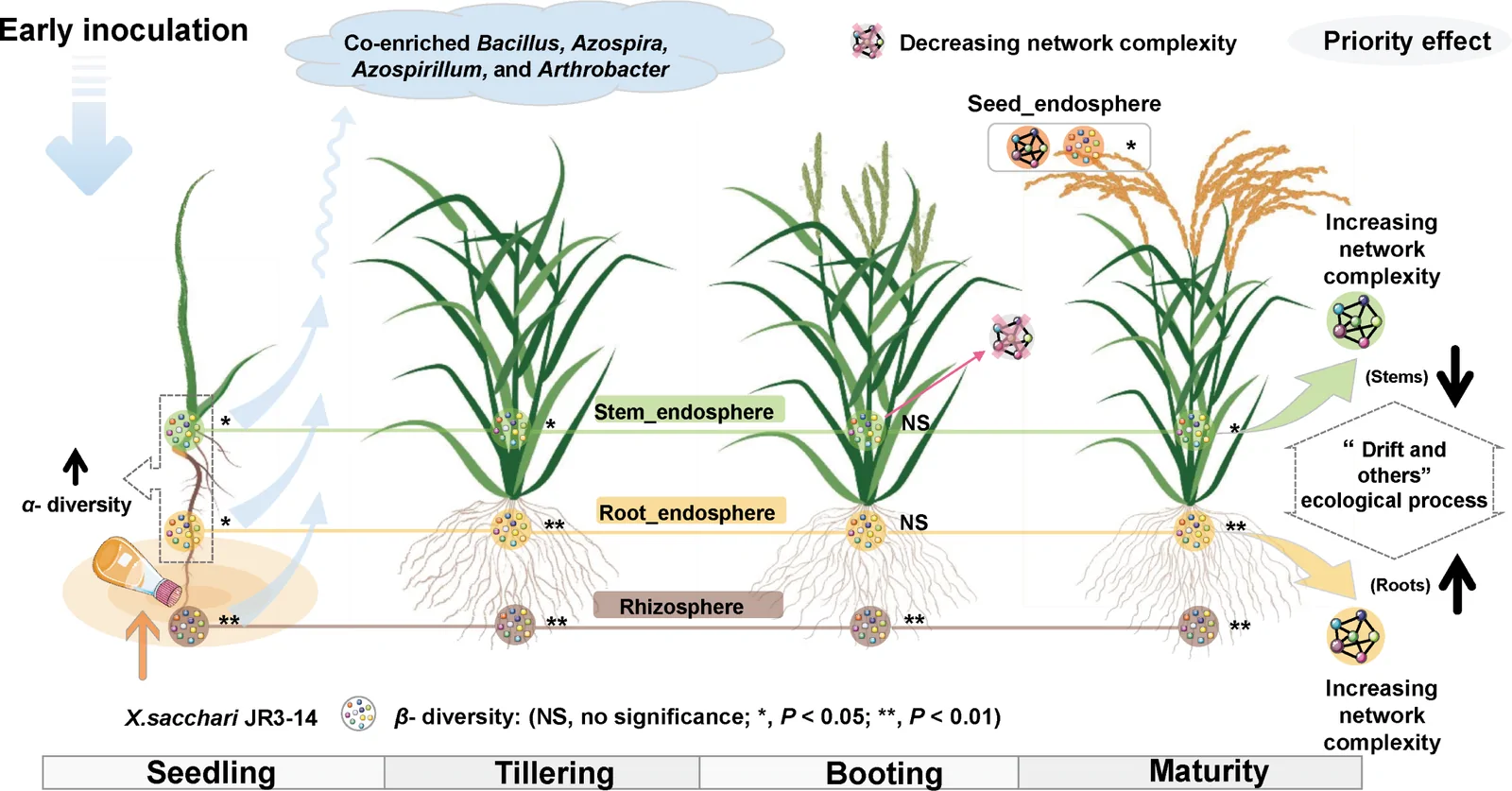
A conceptual model of the impact of early inoculation on microbiomes assembly and ecological processes over the developmental stages in the rice.

Our research indicated that early inoculation significantly increased α-diversity (Shannon index) of the root (*P* < 0.001) and stem endophytic compartments (*P* < 0.05) at the seedling stage, but no significant difference was observed in the rhizosphere during this rice growth stage (Fig. 6). It is accepted that soil microbial communities represent a huge reservoir of biological diversity (50 – 53). The rhizosphere is a narrow zone of soil that can contain up to 10 billion microbial cells per gram root (54) and more than 30,000 prokaryotic species (55). As soil contains a rich and diverse microbiota, this may be why early inoculation of a single endophytic strain did not increase rhizosphere microbial α-diversity at the seedling stage.

Despite early inoculation not causing an increase in rhizosphere community α-diversity, we found in comparison with CK that the microbial composition of the rhizosphere was dominated by Gammaproteobacteria at the seedling stage (Fig. 1a). Gammaproteobacteria were also observed in the root and stem at the tillering stage, indicating that early inoculation changed microbiome composition. Gammaproteobacterial members have the capability to colonize a wide range of ecological niches, such as phyllosphere and rhizosphere, and to play a critical role in regulating host fitness, pathogen suppression, and plant tolerance (56, 57). The results suggest that early inoculation mainly enriched dominant Gammaproteobacteria species in the rhizosphere, root, and stem at the beginning of plant growth.

Furthermore, we performed a comparison analysis to assess specific recruited taxa at the levels of genus. Our results indicated that four genera were co-enriched among the rhizosphere, root endosphere, and stem endosphere compartments, including *Bacillus*, *Azospira*, *Azospirillum*, and *Arthrobacter* (Fig. 6). After predicting the ecological function of bacteria using FAPROTAX analysis, we found that the Xs sample has potential chemoheterotrophy, ureolysis, nitrate_respiration, nitrate_reduction, and aerobic_chemoheterotrophy (Fig. S3). Accordingly, the co-enriched *Azospirillum* and *Azospira* are well-known nitrogen-fixing bacteria (58, 59) and plant growth-promoting bacteria (60 – 62). A recent study showed that *Azospira* could inhibit bacterial wilt disease after inoculation with three *Pseudomonas* strains by enhancing the systemic resistance of tobacco plants (63). *Arthrobacter* has been reported as part of the core microbiota for many plants, including rice (64), lettuce (65), and indoor ornamentals (66). *Bacillus* is one of the dominant endophytic bacteria in seeds of plants such as wheat (67), *Arabidopsis* (68), rice (23), barley (8), and maize (69). They are often recognized as potential plant growth-promoting bacteria (70, 71). Seed-transmitted *Arthrobacter* spp. have been documented for their antifungal activities against the destructive rice pathogens *Rhizoctonia solani* and *Pyricularia grisea* (72). In conclusion, a search of the published literature and functional annotation of bacterial communities in the current study indicated that the majority of taxa recruited at the seedling stage were beneficial to the growth of rice plants. However, in the current research, we have not yet isolated these bacteria and further studies are needed to culture them and examine their biological functions.

It is well-known that multivariate statistical techniques, such as clustering and sequencing, are used to describe β-diversity patterns, revealing how biological and abiotic variables control microbial community composition (73 – 75). In our research, early inoculation altered the bacterial community β-diversity in root, stem, seed and rhizosphere compartments during rice growth except for the roots and stems at the booting stage (Fig. 6); these results suggested that early inoculation had a great impact on microbial diversity. Several studies have shown that endophytic bacteria change dynamically at different developmental stages from seed formation, maturation to germination, as well as the composition and community (76); especially with the development of seeds, the accumulation of starch and other nutrients will affect the composition of seed endophytes and epiphytes (77 – 79). This indicates that after early inoculation, the selective preferences of seeds toward microorganisms may result in differences in β-diversity during booting stage.

It has been suggested that an important network topological feature is the average degree, with a higher average degree representing a greater network complexity (80 – 82). Our results showed that the network structure complexity and positive interconnections of Xs were higher than that of CK, with exception of the stem endosphere at the booting stage (Fig. 6). Generally, the positive correlation connections in the co-occurrence network represent mutualistic relationships between microorganisms, while the negative correlation connections represent potential antagonistic effects (83 – 85). A recent study has shown that inoculation with the endophytic bacterium *Bacillus subtilis* N-1-gfp at seedling stage has a positive effect on the bacterial community associated with rice and also has the ability to promote plant growth (86). The inoculation of *Lactobacillus plantarum* strains has an impact on bacterial community successions during the early and middle ensiling periods of corn, including the complexity of network structure (87). Therefore, it can be seen that early inoculation with a single strain alter the microbial community structure and even increase the complexity of the network, which is also reflected in our research results.

Finally, we assessed and compared the relative contributions of each assembly process of the early inoculated and uninoculated treatments in the rhizosphere, root endosphere, and stem endosphere. In this work, the results from iCAMP indicated that the relative importance of the “drift and others” process was higher in Xs than in CK in the root compartment. Conversely, in the stem compartment, the relative importance of “drift and others” process was lower in Xs (Fig. 6). According to iCAMP analysis, the “drift and others” process consists of weak selection, weak dispersal, diversification, and/or drift (42, 44). It suggests that the boundaries between priority effect, drift, and diversification ecological processes in the quantitative framework were indistinguishable but that the relative contribution of any single part, especially that of priority effect process, could not be neglected in this work. Priority effects refer to small differences in early colonization (such as the order of species arrival) that can result in large differences in community structure in plant microbiome assembly (17, 88). The mechanism of priority effects can be divided into niche preemption or niche modification (88). The concept of priority effects has been applied to investigate the critical role of early interventions on community assembly in leaves (89, 90), roots (91), and rhizospheres (92) based on the persistence of a microbe (core) and/or its importance on microbial networks (hub) and/or SynCom (synthetic communities). A recent study showed that the independent inoculation of over 200 soil slurries on the surface of wheat seeds affected the assembly of microbial communities in 7-day-old wheat seedlings (93). However, our work mainly highlights that early inoculation with a seed endophytic strain has significant effects on later microbiome states. Therefore, the results obtained from this work, including the effects on microbial community assembly, increasing the α-diversity, positive correlation and the complexity of the network structure, recruitment of abundant beneficial bacteria, and changing β-diversity were speculated to be mediated by the priority-effect ecological process.

## MATERIALS AND METHODS

### Source of strain, rice cultivation, experimental design, sample collection, and sample processing

*X. sacchari* JR3-14 (Xs)was isolated from rice (cultivar, Dular) seeds and maintained in our lab (whole-genome accession number is CP099534) (37). Sterile tissue culture seedlings of rice were obtained from the Institute of Crop Science (Chinese Academy of Agricultural Sciences, Beijing). When transplanting sterile cultured seedlings, JR3-14 was inoculated at a concentration of 10^8^ CFU/mL and soaked for 30 min before being inoculated into the roots of rice plants. The control (CK) was treated an equal amount of sterilized medium (tryptone, 10 g/L; yeast extract, 5 g/L; NaCl, 10 g/L), six pots per treatment, six plants per pot.

The samples were collected at the seedling stage (15 days after the inoculation of Xs), tillering stage (40 days after the inoculation of Xs), booting stage (75 days after the inoculation of Xs), and maturity stage (105 days after the inoculation of Xs), including rhizosphere soil, roots, and stems. Bulk soil material was sampled from the six pots far from rhizosphere soil at the seedling stage in Xs and CK treatments. Seeds were collected after harvest. The root-associated compartments were separated into root endosphere and rhizosphere as previously described (5). The entire root clump is shaken to remove loose soil, and a 1-2 millimeter thick layer of soil tightly attached to the roots is collected, and then stored at -80℃ until further processing. Husked seeds, root, and stem tissues were surface sterilized by sequential washing with ultrasonic cleaning using phosphate-buffered saline (PBS; 7 mM Na_2_HPO_4_, 3 mM NaH_2_PO_4_, with 200 mL Tween 20, to 1 L with sterile water) lasting for 2 s with pauses of 1 s, for a total of 5 min, with a power of 237.5 W (950 W * 35%), and finally rinsed with sterile distilled water three times. The ultrasonicated tissues were soaked in 3% (vol/vol) NaClO for 2 min, again, 70% ethanol for 30 s, and washed three times with sterile water. After surface sterilization, all samples were stored at −80°C until DNA was extracted.

### DNA extraction and 16s rRNA gene sequencing

DNA extractions were performed from 500 mg samples, bulk soils (8 samples), rhizosphere soils (48 samples), surface sterilized roots (44 samples), surface sterilized stems (38 samples), and surface sterilized seeds (10 samples) using the FastDNA SPIN Kit for Soil (MP Biomedicals, USA) procedure. DNA quality and concentration were quantified by NanoDrop spectrophotometers (Thermo Scientific, USA). The extracted DNAs were stored at −20°C.

16S rRNA gene sequencing was performed using the Illumina MiSeq platform (Illumina, Inc.) for all samples. This protocol targeted the V3–V4 region of the 16S rRNA gene by using primers 534F (5´-CCAGCAGCCGCGGTAAT-3´) and 783R (5´-ACCMGGGTATCTAATCCKG-3´) (94). The PCR reaction mix contained 25 µL 2× Taq PCR StarMix with Loading Dye (Genstar, Beijing, China), 2 µL of both forward and reverse primers (2 µM), 0.2 µL TopTaq DNA Polymerase (Qiagen) (5 U µL ^−1^), 3 µL of DNA template, and 18 of ddH_2_O. Amplification was performed with an initial denaturation of 3 min at 95°C, followed by 35 cycles of 45 s at 94°C (denaturation), 60 s at 53°C (annealing), 1 min at 72°C (extension), and a final extension at 72°C for 10 min. Size of the PCR products (250 bp) was confirmed on a 1.2% agarose gel. The PCR products were purified using the Gel Extraction kit (OMEGA Bio-tek, USA) following the manufacturer’s instructions. The purified PCR products were standardized according to the net optical density value as assessed with a NanoDrop spectrophotometers (Thermo Scientific, USA), and each sample was mixed at 150 ng. Paired-end (2 × 250) sequencing was performed on the NovaSeq platform (Illumina, San Diego, CA, USA) by Novogene Biotechnology Co., Ltd (Beijing, China).

### Bioinformatics and microbial community analysis

The sequences were demultiplexed to each forward and reverse samples according to the unique barcodes and the primers removed using cutadapt by QIIME2 (95). Paired reads were merged using USEARCH V.10 (96). The resultant sequences were quality filtered (240 bp < length < 260 bp, avg 248 bp), singletons were removed, and dereplication was conducted in VSEARCH V2.7.0 (97). All correct biological reads (i.e., zero-radius operational taxonomic units) were picked using Unoise3 (98) with default parameters in USEARCH V.10. Sequences were assigned ZOTUs at a 97% sequence similarity threshold. Taxonomy was assigned to ZOTUs using the SINTAX algorithm (99) with a 50% confidence threshold by rdp_16 s_v16_sp for 16S rRNA gene in VSEARCH V2.7.0. Sequences from host DNA and ZOTUs unassigned to the bacteria were removed (“Archaea,” “Mitochondria,” “Chloroplast,” and “Unassigned”), specifically, phylum-level “unassigned” were removed. The ZOTUs table was rarefied to 872 reads (lowest number in seed endosphere) for processing estimates among different niches.

Bacterial β-diversity was assessed by computing Bray-Curtis dissimilarity and then ordinated using PCoA. The significance of different factors on community dissimilarity was tested with PERMANOVA using the “adonis” function of the vegan package in R version 4.1.1 (100). The Shannon and richness index was calculated using vegan package in R. α-Diversity was analyzed using analysis of variance (ANOVA), and one-way ANOVA (*P* < 0.05) and Tukey’s Honestly Significant Difference (HSD) test was performed with SPSS software (SPSS 20.0 for Windows, IBM Corp., Armonk, NY, USA). Statistical visualization was generated using ggplot2 package in R version 4.1.1 (101). All histograms were generated with GraphPad Prism 8.3.0 (GraphPad Software, San Diego, CA, USA, www.graphpad.com). Differential abundance analysis was used to identify the enrichment of genus compared with CK among the different compartments (R package deseq2) (102). Genera were considered enriched if they had a log_2_ fold change greater than 2 and an adjusted *P*-value less than 0.05. Network analysis was performed using SparCC algorithm to infer co-occurrence patterns. Only the nodes with correlation coefficient (*r* >0.6 significant at *P* < 0.05) were considered, and were visualized with Gephi (v0.9.2) (103). The nodes are colored according to phylum. Blue edges represent positive correlations and red edges represent negative correlations. Node size is proportional to the average degree of each species. The node, edge, and network properties (average degree, connectance, average path distance, and average clustering coefficients) were investigated using igraph (R package). Bacterial ZOTUs were assigned to multiple functional groups using FAPROTAX v.1.2.4 (104).

The community assembly mechanism was quantitatively inferred using phylogenetic bin-based null model analysis (iCAMP), which was recently reported with substantially improved performance (44). Briefly, iCAMP divided taxa into different phylogenetic groups (bins) to ensure adequate phylogenetic signal to infer selection from phylogenetic diversity. For each bin, the fraction of pairwise comparisons with beta net relatedness index (βNRI) <−1.96 is considered as the percentages of homogeneous selection, whereas those with βNRI >+1.96 is considered as the percentages of heterogeneous selection. Next, taxonomic diversity metric Raup-Crick metric (RC) is used to partition the remaining pairwise comparisons with |βNRI|  ≤  1.96. RC <−0.95 represents homogenizing dispersal, while those with RC >+0.95 as dispersal limitation. The remains with |βNRI|  ≤  1.96 and |RC|  ≤  0.95 represent the percentages of drift and others. In short, FastTree is used to build a phylogenetic tree based on aligned sequences (105, 106). The "iCAMP version 1.3.2" and the Galaxy platform pipeline (http://ieg3.rccc.ou.edu:8080) are used with default settings to compute and determine the relative importance of each ecological process.

### Conclusion

In this study, we provide comprehensive and empirical evidence on the relative contribution of early inoculation with an endophytic core bacterium to microbiome assembly in rice. Our results demonstrate that microbiome composition from rhizosphere to various plant compartments significantly enriched Gammaproteobacteria in rhizosphere at the seedling stage, and in root and stem compartments at the tillering stage. Moreover, we provide empirical evidence that microbiome assembly (β-diversity) over the developmental stages of rice in each compartment is altered by early inoculation, with exception of stems and roots samples at the booting stage. Furthermore, we revealed that early inoculation had a strong effect on increasing bacterial α-diversity in root and stem compartments at the seedling stage, and enhancing network complexity and positive correlation of network structure from most seedling stage to maturity stage in endosphere samples. *X. sacchari* may be able to recruit some beneficial taxa from the nearby/local species pool. Four abundant genera (*Bacillus*, *Azospira*, *Azospirillum*, *Arthrobacter*) were significantly co-enriched at the seedling stage in this study. Finally, our study highlights the important role of the “drift and others” ecology process in governing the microbial community assembly of root and stem.

## Data Availability

Scripts for the bioinformatic analysis and plotting used in this study are available at https://github.com/wangxing1997/Early-inoculated-an-endophyte20230406.git. Raw sequence data for 16S rRNA gene amplicons are available in the NCBI Sequence Read Archive (SRA) under accession number PRJNA836034. Whole Genome data of *Xanthomonas sacchair* JR3-14 has been deposited at DDBJ/ENA/GenBank under the accession CP099534.

## SUPPLEMENTAL MATERIAL

Figure S1 spectrum.04978-22-s0001.tif Relative abundances of the most abundant phylum level in seed endosphere and bulk soil between Xs and CK.

Figure S2 spectrum.04978-22-s0002.tif Co-occurrence network in seed compartment.

Figure S3 spectrum.04978-22-s0003.tif Heat map of metabolic and ecological functions of bacteria.

Supplemental legends spectrum.04978-22-s0004.docx Legends to supplemental material.

Table S1 spectrum.04978-22-s0005.xlsx Strain information.

Table S2 spectrum.04978-22-s0006.xlsx Enrichment and depletion patterns.

ASM does not own the copyrights to Supplemental Material that may be linked to, or accessed through, an article. The authors have granted ASM a non-exclusive, world-wide license to publish the Supplemental Material files. Please contact the corresponding author directly for reuse.
