# Supplementary figures and images for: Early inoculation of an endophyte alters the assembly of bacterial communities across rice plant growth stages
Source: Microbiol Spectr. 2023 Sep 1;11(5):e04978-22. doi: 10.1128/spectrum.04978-22 (PMC10580921; doi:10.1128/spectrum.04978-22)

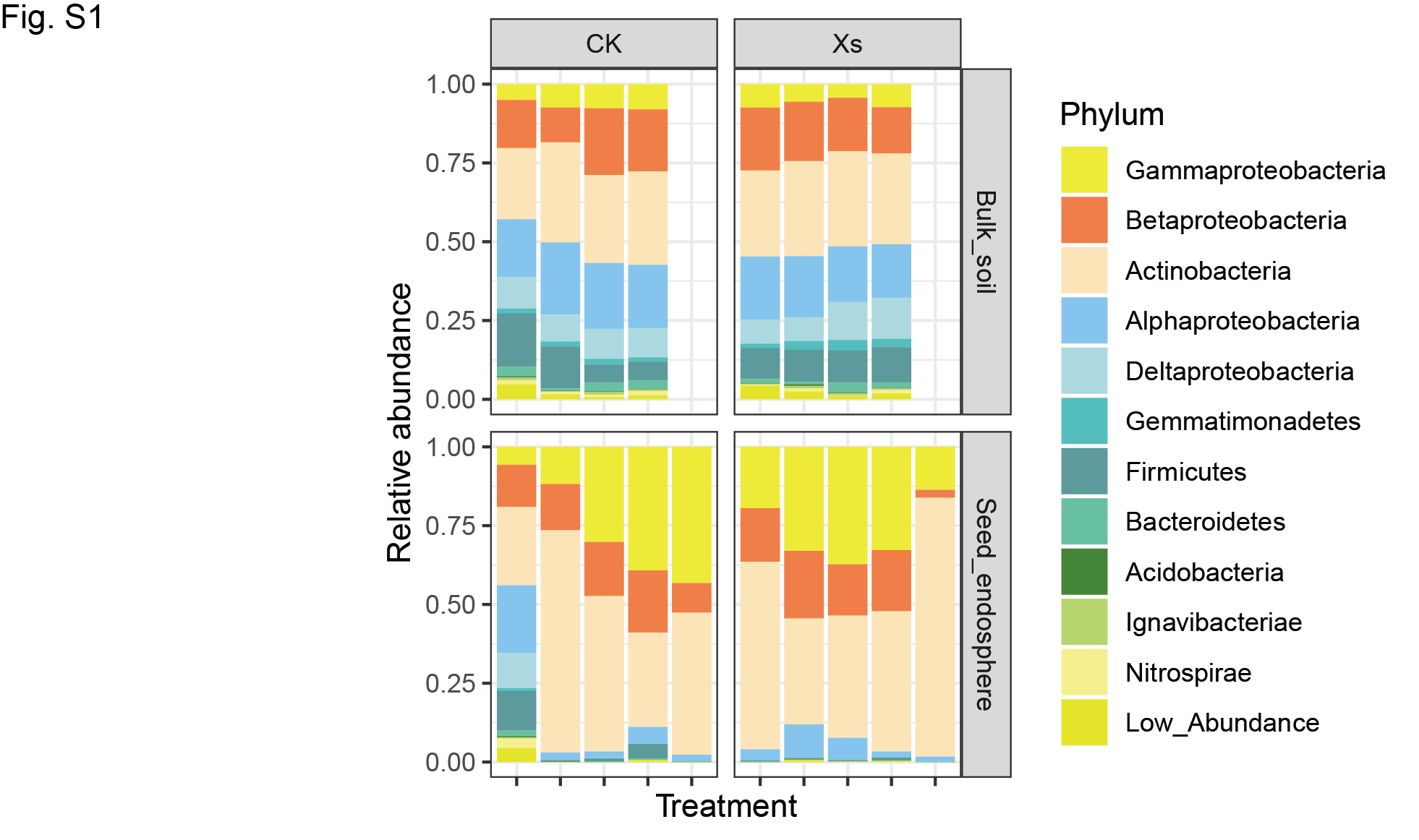

Supplement: Figure S1 — Relative abundances of the most abundant phylum level in seed endosphere and bulk soil between Xs and CK. [file spectrum.04978-22-s0001.tif]

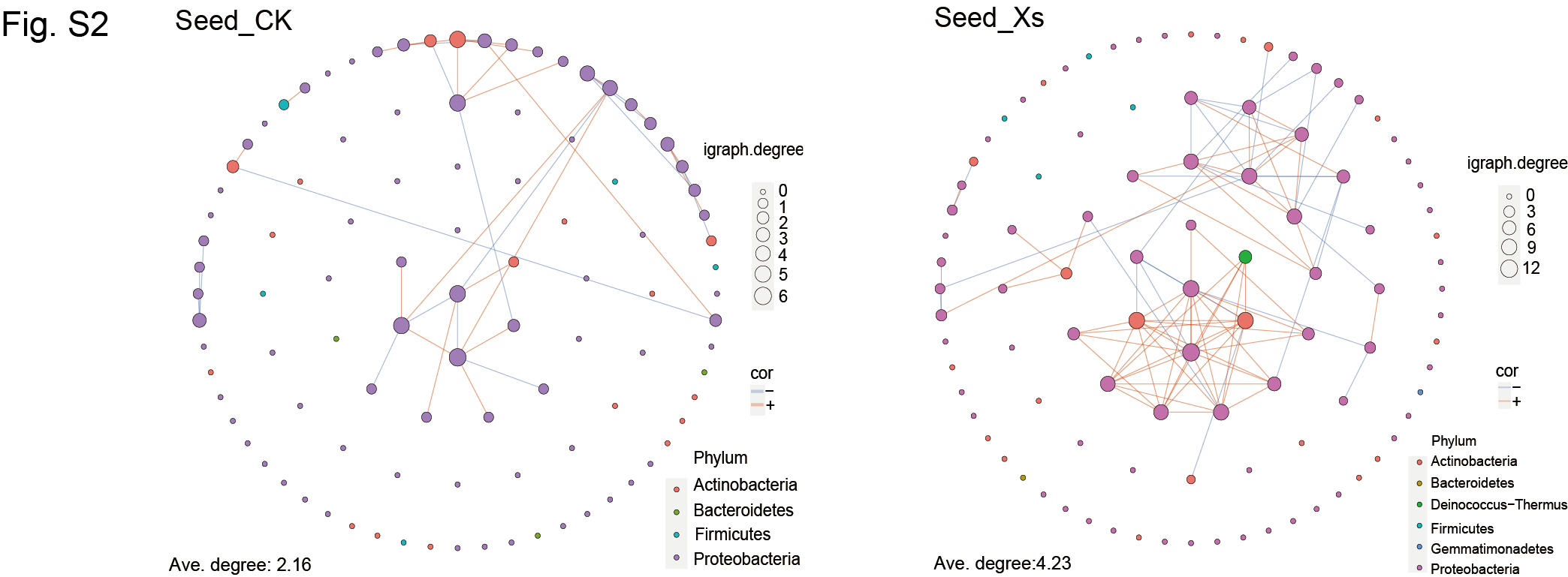

Supplement: Figure S2 — Co-occurrence network in seed compartment. [file spectrum.04978-22-s0002.tif]

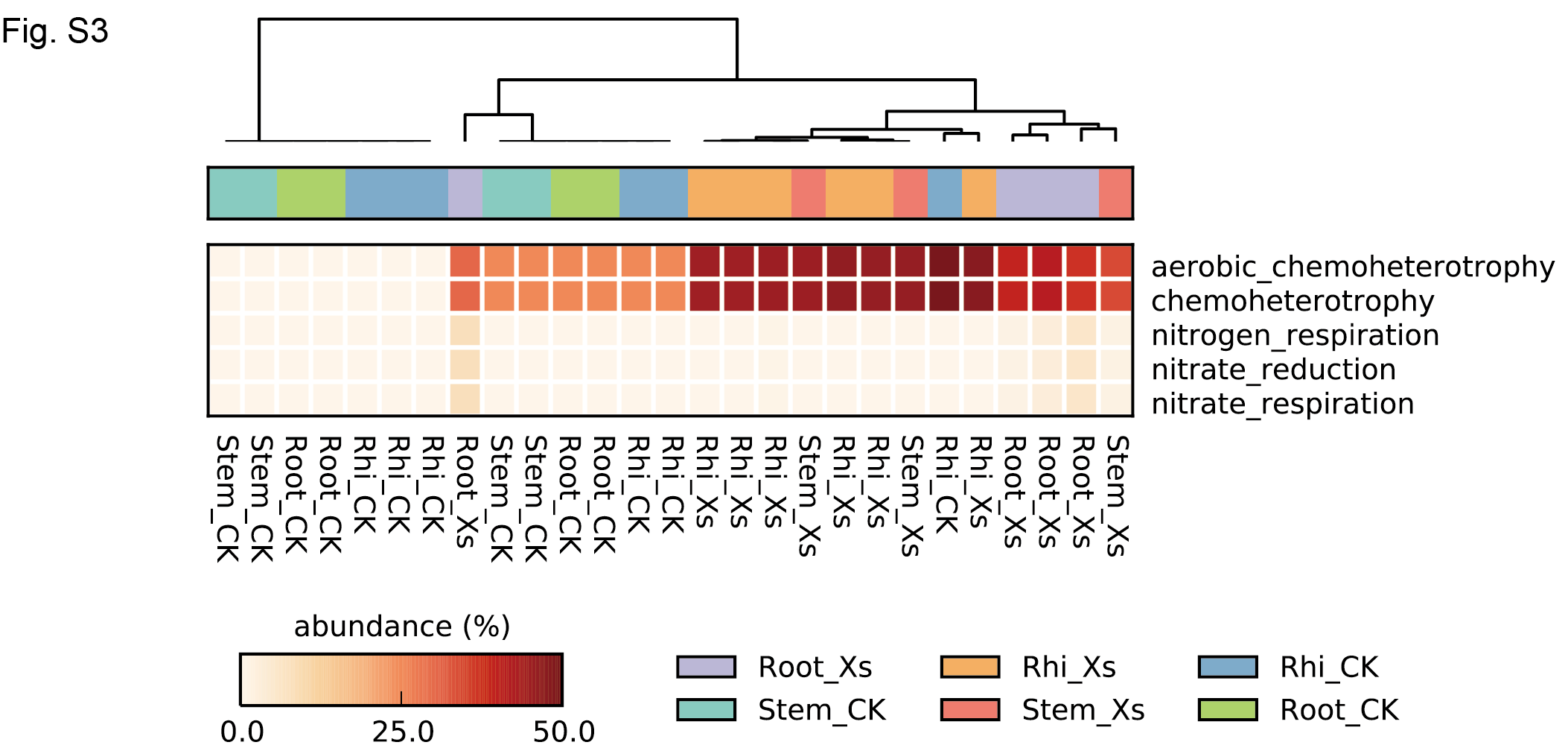

Supplement: Figure S3 — Heat map of metabolic and ecological functions of bacteria. [file spectrum.04978-22-s0003.tif]
