# Supplementary material for: Early inoculation of an endophyte alters the assembly of bacterial communities across rice plant growth stages
Source: Microbiol Spectr. 2023 Sep 1;11(5):e04978-22. doi: 10.1128/spectrum.04978-22 (PMC10580921; doi:10.1128/spectrum.04978-22)
Supplement: Supplemental legends — Legends to supplemental material. [file spectrum.04978-22-s0004.docx]

**Fig. S1** The relative abundances of the most abundant phylum level in seed endosphere and bulk soil between Xs and CK. Proteobacteria is presented as (sub)phylum, which includes Alphaproteobacteria, Gammaproteobacteria, Betaproteobacteria, Deltaproteobacteria.

**Fig. S2** Co-occurrence network in seed compartment. SparCC algorithm was used to calculate the network at the ZOTU level with r > 0.6 and p < 0,05. Each node size represents average degree. Color of edges indicates the type of the interaction. Red, negative; blue, positive.

**Fig. S3** Heatmap of metabolic and ecological functions of bacteria based on FAPROTAX prediction in rhizosphere, roots, and stems at the seedling stage.

**Table S1** Strains information based on the high similarity of 16S rRNA gene with *X. sacchari* in NCBI database.

**Table S2** The enrichment and depletion patterns of the early inoculation in each compartment niche at the four stages.

**Reference**

1. Ali M, Ali Q, Sohail MA, Ashraf MF, Saleem MH, Hussain S, Zhou L. 2021. Diversity and taxonomic distribution of endophytic bacterial community in the rice plant and its prospective. Int J Mol Sci 22:10165. https://doi.org/10.3390/IJMS221810165.

2. Santoyo G, Moreno-Hagelsieb G, Orozco-Mosqueda Mdel C, Glick BR. 2016. Plant growth-promoting bacterial endophytes. Microbiol Res 183:92-99. https://doi.org/10.1016/j.micres.2015.11.008.

3. Rosenblueth M, Martínez-Romero E. 2006. Bacterial endophytes and their interactions with hosts. Mol Plant-Microbe Interact 19:827-837. https://doi.org/10.1094/mpmi-19-0827.

4. Fitzpatrick CR, Copeland J, Wang PW, Guttman DS, Kotanen PM, Johnson M. 2018. Assembly and ecological function of the root microbiome across angiosperm plant species. PNAS 115:E1157-e1165. https://doi.org/10.1073/pnas.1717617115.

5. Edwards J, Johnson C, Santos-Medellín C, Lurie E, Podishetty NK, Bhatnagar S, Eisen JA, Sundaresan V. 2015. Structure, variation, and assembly of the root-associated microbiomes of rice. PNAS 112:E911-E920. https://doi.org/10.1073/pnas.1414592112.

6. Berg G, Raaijmakers JM. 2018. Saving seed microbiomes. ISME J 12:1167-1170. https://doi.org/10.1038/s41396-017-0028-2.

7. Nelson EB. 2018. The seed microbiome: Origins, interactions, and impacts. Plant Soil 422:7-34. https://doi.org/10.1007/s11104-017-3289-7.

8. Rahman MM, Flory E, Koyro HW, Abideen Z, Schikora A, Suarez C, Schnell S, Cardinale M. 2018. Consistent associations with beneficial bacteria in the seed endosphere of barley (*Hordeum vulgare* L.). Syst Appl Microbiol 41:386-398. https://doi.org/10.1016/j.syapm.2018.02.003.

9. Sánchez-López AS, Thijs S, Beckers B, González-Chávez MC, Weyens N, Carrillo-González R, Vangronsveld J. 2018. Community structure and diversity of endophytic bacteria in seeds of three consecutive generations of *Crotalaria pumila* growing on metal mine residues. Plant Soil 422:51-66. https://doi.org/10.1007/s11104-017-3176-2.

10. Shade A, Jacques MA, Barret M. 2017. Ecological patterns of seed microbiome diversity, transmission, and assembly. Curr Opin Microbiol 37:15-22. https://doi.org/10.1016/j.mib.2017.03.010.

11. Hardoim PR, van Overbeek LS, Berg G, Pirttila AM, Compant S, Campisano A, Doring M, Sessitsch A. 2015. The hidden world within plants: ecological and evolutionary considerations for defining functioning of microbial endophytes. Microbiol Mol Biol Rev 79:293-320. https://doi.org/10.1128/mmbr.00050-14.

12. Truyens S, Weyens N, Cuypers A, Vangronsveld J. 2015. Bacterial seed endophytes: genera, vertical transmission and interaction with plants. Environ Microbiol Rep 7:40-50. https://doi.org/10.1111/1758-2229.12181.

13. Hardoim PR, Hardoim CC, van Overbeek LS, van Elsas JD. 2012. Dynamics of seed-borne rice endophytes on early plant growth stages. PLoS One 7:e30438. https://doi.org/10.1371/journal.pone.0030438.

14. Kaga H, Mano H, Tanaka F, Watanabe A, Kaneko S, Morisaki H. 2009. Rice seeds as sources of endophytic bacteria. Microbes Environ 24:154-162. https://doi.org/10.1264/jsme2.ME09113.

15. White JF, Kingsley KI, Kowalski KP, Irizarry I, Micci A, Soares MA, Bergen MS. 2018. Disease protection and allelopathic interactions of seed-transmitted endophytic pseudomonads of invasive reed grass (*Phragmites australis*). Plant Soil 422:195-208. https://doi.org/10.1007/s11104-016-3169-6.

16. Shao JH, Miao YZ, Liu KM, Ren Y, Xu ZH, Zhang N, Feng HC, Shen QR, Zhang RF, Xun WB. 2021. Rhizosphere microbiome assembly involves seed-borne bacteria in compensatory phosphate solubilization. Soil Biol Biochem 159:108273. https://doi.org/10.1016/j.soilbio.2021.108273.

17. Toju H, Peay KG, Yamamichi M, Narisawa K, Hiruma K, Naito K, Fukuda S, Ushio M, Nakaoka S, Onoda Y, Yoshida K, Schlaeppi K, Bai Y, Sugiura R, Ichihashi Y, Minamisawa K, Kiers ET. 2018. Core microbiomes for sustainable agroecosystems. Nat Plants 4:247-257. https://doi.org/10.1038/s41477-018-0139-4.

18. Hacquard S, Garrido-Oter R, González A, Spaepen S, Ackermann G, Lebeis S, McHardy AC, Dangl JL, Knight R, Ley R, Schulze-Lefert P. 2015. Microbiota and host nutrition across plant and animal kingdoms. Cell Host Microbe 17:603-616. https://doi.org/10.1016/j.chom.2015.04.009.

19. Kim H, Lee KK, Jeon J, Harris WA, Lee YH. 2020. Domestication of Oryza species eco-evolutionarily shapes bacterial and fungal communities in rice seed. Microbiome 8:20. https://doi.org/10.1186/s40168-020-00805-0.

20. Walitang DI, Kim CG, Jeon S, Kang Y, Sa T. 2019. Conservation and transmission of seed bacterial endophytes across generations following crossbreeding and repeated inbreeding of rice at different geographic locations. MicrobiologyOpen 8:e00662. https://doi.org/10.1002/mbo3.662.

21. Wang M, Eyre AW, Thon MR, Oh Y, Dean RA. 2020. Dynamic changes in the microbiome of rice during shoot and root growth derived from seeds. Front Microbiol 11:559728. https://doi.org/10.3389/fmicb.2020.559728.

22. Kim H, Jeon J, Lee KK, Lee YH. 2022. Longitudinal transmission of bacterial and fungal communities from seed to seed in rice. Commun Biol 5:772. https://doi.org/10.1038/s42003-022-03726-w.

23. Zhang J, Zhang CW, Yang J, Zhang RJ, Gao JS, Zhao X, Zhao JJ, Zhao DF, Zhang XX. 2019. Insights into endophytic bacterial community structures of seeds among various *Oryza sativa* L. rice genotypes. J Plant Growth Regul 38:93-102. https://doi.org/10.1007/s00344-018-9812-0.

24. Jacques MA, Arlat M, Boulanger A, Boureau T, Carrere S, Cesbron S, Chen NWG, Cociancich S, Darrasse A, Denance N, Fischer-Le Saux M, Gagnevin L, Koebnik R, Lauber E, Noel LD, Pieretti I, Portier P, Pruvost O, Rieux A, Robene I, Royer M, Szurek B, Verdier V, Verniere C. 2016. Using ecology, physiology, and genomics to understand host specificity in *Xanthomonas*. Annu Rev Phytopathol 54:163-187. https://doi.org/10.1146/annurev-phyto-080615-100147.

25. Timilsina S, Potnis N, Newberry EA, Liyanapathiranage P, Iruegas-Bocardo F, White FF, Goss EM, Jones JB. 2020. *Xanthomonas* diversity, virulence and plant-pathogen interactions. Nat Rev Microbiol 18:415-427. https://www.ncbi.nlm.nih.gov/pubmed/32346148.

26. Boureau T, Kerkoud M, Chhel F, Hunault G, Darrasse A, Brin C, Durand K, Hajri A, Poussier S, Manceau C, Lardeux F, Saubion F, Jacques MA. 2013. A multiplex-PCR assay for identification of the quarantine plant pathogen *Xanthomonas axonopodis* pv. *phaseoli*. J Microbiol Methods 92:42-50. https://doi.org/10.1016/j.mimet.2012.10.012.

27. Cesbron S, Briand M, Essakhi S, Gironde S, Boureau T, Manceau C, Fischer-Le Saux M, Jacques MA. 2015. Comparative genomics of pathogenic and nonpathogenic strains of *Xanthomonas arboricola* unveil molecular and evolutionary events linked to pathoadaptation. Front Plant Sci 6:1126. https://doi.org/10.3389/fpls.2015.01126.

28. Essakhi S, Cesbron S, Fischer-Le Saux M, Bonneau S, Jacques MA, Manceau C. 2015. Phylogenetic and variable-number tandem-repeat analyses identify nonpathogenic *Xanthomonas arboricola* lineages lacking the canonical type III secretion system. Appl Environ Microbiol 81:5395-5410. https://doi.org/10.1128/AEM.00835-15.

29. Fang Y, Lin H, Wu L, Ren D, Ye W, Dong G, Zhu L, Guo L. 2015. Genome sequence of *Xanthomonas sacchari* R1, a biocontrol bacterium isolated from the rice seed. J Biotechnol 206:77-78. https://doi.org/10.1016/j.jbiotec.2015.04.014.

30. Garita-Cambronero J, Palacio-Bielsa A, Lopez MM, Cubero J. 2016. Comparative genomic and phenotypic characterization of pathogenic and non-pathogenic strains of *Xanthomonas arboricola* reveals insights into the infection process of bacterial spot disease of stone fruits. PLoS One 11:e0161977. https://doi.org/10.1371/journal.pone.0161977.

31. Bansal K, Kaur A, Midha S, Kumar S, Korpole S, Patil PB. 2021. *Xanthomonas sontii* sp. nov., a non-pathogenic bacterium isolated from healthy basmati rice (*Oryza sativa*) seeds from India. Antonie van Leeuwenhoek 114:1935–1947. https://doi.org/10.1007/s10482-021-01652-1.

32. Kay S, Bonas U. 2009. How *Xanthomonas* type III effectors manipulate the host plant. Curr Opin Microbiol 12:37-43. https://doi.org/10.1016/j.mib.2008.12.006.

33. Shrivastava S, Mande SS. 2008. Identification and functional characterization of gene components of Type VI Secretion system in bacterial genomes. PLoS One 3:e2955. https://doi.org/10.1371/journal.pone.0002955.

34. Pal G, Kumar K, Verma A, White JF, Verma SK. 2019. Functional roles of seed-inhabiting endophytes of rice. Seed Endophytes: Biology and Biotechnology p 213-236. https://doi.org/10.1007/978-3-030-10504-4_11.

35. Raj G, Shadab M, Deka S, Das M, Baruah J, Bharali R, Talukdar NC. 2019. Seed interior microbiome of rice genotypes indigenous to three agroecosystems of Indo-Burma biodiversity hotspot. BMC Genom 20:924. https://doi.org/10.1186/s12864-019-6334-5.

36. Walitang DI, Kim K, Madhaiyan M, Kim YK, Kang Y, Sa T. 2017. Characterizing endophytic competence and plant growth promotion of bacterial endophytes inhabiting the seed endosphere of Rice. BMC Microbiol 17:209. https://doi.org/10.1186/s12866-017-1117-0.

37. Zhang X, Ma YN, Wang X, Liao K, He S, Zhao X, Guo H, Zhao D, Wei HL. 2022. Dynamics of rice microbiomes reveal core vertically transmitted seed endophytes. Microbiome 10:216. https://doi.org/10.1186/s40168-022-01422-9.

38. Hassani MA, Durán P, Hacquard S. 2018. Microbial interactions within the plant holobiont. Microbiome 6:58. https://doi.org/10.1186/s40168-018-0445-0.

39. Vandenkoornhuyse P, Quaiser A, Duhamel M, Le Van A, Dufresne A. 2015. The importance of the microbiome of the plant holobiont. New Phytol 206:1196-1206. https://doi.org/10.1111/nph.13312.

40. Cordovez V, Dini-Andreote F, Carrión VJ, Raaijmakers JM. 2019. Ecology and evolution of plant microbiomes. Annu Rev Microbiol 73:69-88. https://doi.org/10.1146/annurev-micro-090817-062524.

41. Fitzpatrick CR, Salas-González I, Conway JM, Finkel OM, Gilbert S, Russ D, Teixeira P, Dangl JL. 2020. The plant microbiome: from ecology to reductionism and beyond. Annu Rev Microbiol 74:81-100. https://doi.org/10.1146/annurev-micro-022620-014327.

42. Zhou J, Ning D. 2017. Stochastic community assembly: does it matter in microbial ecology? Microbiol Mol Biol Rev 81:e00002-17. https://doi.org/10.1128/mmbr.00002-17.

43. Ning D, Deng Y, Tiedje JM, Zhou J. 2019. A general framework for quantitatively assessing ecological stochasticity. PNAS 116:16892-16898. https://doi.org/10.1073/pnas.1904623116.

44. Ning D, Yuan M, Wu L, Zhang Y, Guo X, Zhou X, Yang Y, Arkin AP, Firestone MK, Zhou J. 2020. A quantitative framework reveals ecological drivers of grassland microbial community assembly in response to warming. Nat Commun 11:4717. https://doi.org/10.1038/s41467-020-18560-z.

45. Sun C, Zhang B, Ning D, Zhang Y, Dai T, Wu L, Li T, Liu W, Zhou J, Wen X. 2021. Seasonal dynamics of the microbial community in two full-scale wastewater treatment plants: Diversity, composition, phylogenetic group based assembly and co-occurrence pattern. Water Res 200:117295. https://doi.org/10.1016/j.watres.2021.117295.

46. Sun Y, Zhang M, Duan C, Cao N, Jia W, Zhao Z, Ding C, Huang Y, Wang J. 2021. Contribution of stochastic processes to the microbial community assembly on field-collected microplastics. Environ Microbiol 23:6707-6720. https://doi.org/10.1111/1462-2920.15713.

47. Yi M, Fang Y, Hu GP, Liu SF, Ni JR, Liu T. 2021. Distinct community assembly processes underlie significant spatiotemporal dynamics of abundant and rare bacterioplankton in the Yangtze River. Front Environ Sci Eng 16:79. https://doi.org/10.1007/s11783-021-1513-4.

48. Xie J, Wang X, Xu J, Xie H, Cai Y, Liu Y, Ding X. 2021. Strategies and structure feature of the aboveground and belowground microbial community respond to drought in wild rice (*Oryza longistaminata*). Rice (New York, NY) 14:79. https://doi.org/10.1186/s12284-021-00522-8.

49. Xiong C, Zhu YG, Wang JT, Singh B, Han LL, Shen JP, Li PP, Wang GB, Wu CF, Ge AH, Zhang LM, He JZ. 2021. Host selection shapes crop microbiome assembly and network complexity. New Phytol 229:1091-1104. https://doi.org/10.1111/nph.16890.

50. Buée M, Reich M, Murat C, Morin E, Nilsson RH, Uroz S, Martin F. 2009. 454 Pyrosequencing analyses of forest soils reveal an unexpectedly high fungal diversity. New Phytol 184:449-456. https://doi.org/10.1111/j.1469-8137.2009.03003.x.

51. Curtis TP, Sloan WT, Scannell JW. 2002. Estimating prokaryotic diversity and its limits. PNAS 99:10494-10499. https://doi.org/10.1073/pnas.142680199.

52. Gams W. 2007. Biodiversity of soil-inhabiting fungi. Biodivers Conserv 16:69-72. https://doi.org/10.1007/s10531-006-9121-y.

53. Torsvik V, Ovreas L, Thingstad TF. 2002. Prokaryotic diversity-magnitude, dynamics, and controlling factors. Science 296:1064-1066. https://doi.org/10.1126/science.1071698.

54. Rosselló-Mora R, Amann R. 2001. The species concept for prokaryotes. FEMS Microbiol Rev 25:39-67. https://doi.org/10.1111/j.1574-6976.2001.tb00571.x.

55. Egamberdieva D, Kamilova F, Validov S, Gafurova L, Kucharova Z, Lugtenberg B. 2008. High incidence of plant growth-stimulating bacteria associated with the rhizosphere of wheat grown on salinated soil in Uzbekistan. Environ Microbiol 10:1-9. https://doi.org/10.1111/j.1462-2920.2007.01424.x.

56. Álvarez-Pérez JMG-G, S. Cobos, R., Olego MÁ, Ibañez A, Díez-Galán A, Garzón-Jimeno E, Coque J. 2017. Use of endophytic and rhizosphere Actinobacteria from grapevine plants to reduce nursery fungal graft infections that lead to young grapevine decline. Appl Environ Microbiol 83:e01564-17. https://doi.org/10.1128/aem.01564-17.

57. Mendes R, Kruijt M, de Bruijn I, Dekkers E, van der Voort M, Schneider JHM, Piceno YM, DeSantis TZ, Andersen GL, Bakker P, Raaijmakers JM. 2011. Deciphering the rhizosphere microbiome for disease-suppressive bacteria. Science 332:1097-1100. https://doi.org/10.1126/science.1203980.

58. Jha PN, Gomaa A, Yanni YG, El-Saadany AY, Stedtfeld TM, Stedtfeld RD, Gantner S, Chai BL, Cole J, Hashsham SA, Dazzo FB. 2020. Alterations in the endophyte-enriched root-associated microbiome of rice receiving growth-promoting treatments of urea fertilizer and rhizobium biofertilizer. Microb Ecol 79:367-382. https://doi.org/10.1007/s00248-019-01406-7.

59. Zhan J, Sun Q. 2011. Diversity of free-living nitrogen-fixing microorganisms in wastelands of copper mine tailings during the process of natural ecological restoration. J Environ Sci 23:476-487. https://doi.org/10.1016/S1001-0742(10)60433-0.

60. García JE, Maroniche G, Creus C, Suárez-Rodríguez RR-T, J. A., Groppa MD. 2017. In vitro PGPR properties and osmotic tolerance of different *Azospirillum* native strains and their effects on growth of maize under drought stress. Microbiol Res 202:21-29. https://doi.org/10.1016/j.micres.2017.04.007.

61. Kaushal M, Wani SP. 2016. Plant-growth-promoting rhizobacteria: drought stress alleviators to ameliorate crop production in drylands. Ann Microbiol 66:35-42. https://doi.org/10.1007/s13213-015-1112-3.

62. Zeffa DM, Perini LJ, Silva MB, de Sousa NV, Scapim CA, de Oliveira AL, do Amaral AT, Goncalves LSA. 2019. *Azospirillum brasilense* promotes increases in growth and nitrogen use efficiency of maize genotypes. PLoS One 14:e0215332. https://doi.org/10.1371/journal.pone.0215332.

63. Shang XC, Cai X, Zhou Y, Han X, Zhang CS, Ilyas N, Li Y, Zheng Y. 2021. *Pseudomonas* inoculation stimulates endophytic *Azospira* population and induces systemic resistance to Bacterial Wilt. Front Plant Sci 12:738611. https://www.frontiersin.org/articles/10.3389/fpls.2021.738611.

64. Zhou X, Wang JT, Zhang ZF, Li W, Chen W, Cai L. 2020. Microbiota in the rhizosphere and seed of rice from China, with reference to their transmission and biogeography. Front Microbiol 11:995. https://doi.org/10.3389/fmicb.2020.00995.

65. Rastogi G, Sbodio A, Tech JJ, Suslow TV, Coaker GL, Leveau JH. 2012. Leaf microbiota in an agroecosystem: spatiotemporal variation in bacterial community composition on field-grown lettuce. ISME J 6:1812-1822. https://doi.org/10.1038/ismej.2012.32.

66. Ortega RA, Mahnert A, Berg C, Müller H, Berg G. 2016. The plant is crucial: specific composition and function of the phyllosphere microbiome of indoor ornamentals. FEMS Microbiol Ecol 92:fiw173. https://doi.org/10.1093/femsec/fiw173.

67. Johnston-Monje D, Gutierrez JP, Lopez-Lavalle LAB. 2021. Seed-transmitted bacteria and fungi dominate juvenile plant microbiomes. Front Microbiol 12:737616. https://doi.org/10.3389/fmicb.2021.737616.

68. Truyens S, Weyens N, Cuypers A, Vangronsveld J. 2013. Changes in the population of seed bacteria of transgenerationally Cd-exposed *Arabidopsis thaliana*. Plant Biology (Stuttgart, Germany) 15:971-981. https://doi.org/10.1111/j.1438-8677.2012.00711.x.

69. Liu Y, Zuo S, Xu L, Zou Y, Song W. 2012. Study on diversity of endophytic bacterial communities in seeds of hybrid maize and their parental lines. Arch Microbiol 194:1001-1012. https://doi.org/10.1007/s00203-012-0836-8.

70. Orozco-Mosqueda MDC, Rocha-Granados MDC, Glick BR, Santoyo G. 2018. Microbiome engineering to improve biocontrol and plant growth-promoting mechanisms. Microbiol Res 208:25-31. https://doi.org/10.1016/j.micres.2018.01.005.

71. Pan FS, Meng Q, Luo S, Shen J, Chen B, Khan KY, Japenga J, Ma XX, Yang XE, Feng Y. 2017. Enhanced Cd extraction of oilseed rape (*Brassica napus*) by plant growth-promoting bacteria isolated from Cd hyperaccumulator Sedum alfredii Hance. Int J Phytorem 19:281-289. https://doi.org/10.1080/15226514.2016.1225280.

72. Cottyn B, Debode J, Regalado E, Mew TW, Swings J. 2009. Phenotypic and genetic diversity of rice seed-associated bacteria and their role in pathogenicity and biological control. J Appl Microbiol 107:885-897. https://doi.org/10.1111/j.1365-2672.2009.04268.x.

73. Auguet JC, Barberan A, Casamayor EO. 2010. Global ecological patterns in uncultured Archaea. ISME J 4:182-190. https://doi.org/10.1038/ismej.2009.109.

74. Barberán A, Casamayor EO. 2011. Euxinic freshwater hypolimnia promote bacterial endemicity in continental areas. Microb Ecol 61:465-472. https://doi.org/10.1007/s00248-010-9775-6.

75. Lozupone CA, Knight R. 2007. Global patterns in bacterial diversity. PNAS 104:11436-11440. https://doi.org/10.1073/pnas.0611525104.

76. Nelson EB. 2004. Microbial dynamics and interactions in the spermospher. Annu Rev Phytopathol 42:271-309. https://doi.org/10.1146/annurev.phyto.42.121603.131041.

77. Mano H, Tanaka F, Watanabe A, Kaga H, Okunishi S, Morisaki H. 2006. Culturable surface and endophytic bacterial flora of the maturing seeds of rice plants (*Oryza sativa*) cultivated in a paddy field. Microbes Environ 21:86-100. https://doi.org/10.1264/jsme2.21.86.

78. Okunishi S, Sako K, Mano H, Imamura A, Morisaki H. 2005. Bacterial flora of endophytes in the maturing seed of cultivated rice (*Oryza sativa*). Microbes Environ 20:168-177. https://doi.org/10.1264/jsme2.20.168.

79. Zhou J, Li P, Meng D, Gu Y, Zheng Z, Yin H, Zhou Q, Li J. 2020. Isolation, characterization and inoculation of Cd tolerant rice endophytes and their impacts on rice under Cd contaminated environment. Environ Pollut 260:113990. https://doi.org/10.1016/j.envpol.2020.113990.

80. Pimm S. 1984. The complexity and stability of ecosystems. Nature 307:321-326. https://doi.org/10.1038/307321a0.

81. Shi S, Nuccio EE, Shi ZJ, He Z, Zhou J, Firestone MK. 2016. The interconnected rhizosphere: High network complexity dominates rhizosphere assemblages. Ecol Lett 19:926-936. https://doi.org/10.1111/ele.12630.

82. Wagg C, Schlaeppi K, Banerjee S, Kuramae EE, van der Heijden MGA. 2019. Fungal-bacterial diversity and microbiome complexity predict ecosystem functioning. Nat Commun 10:4841. https://doi.org/10.1038/s41467-019-12798-y.

83. Berry D, Widder S. 2014. Deciphering microbial interactions and detecting keystone species with co-occurrence networks. Front Microbiol 5. https://doi.org/10.3389/fmicb.2014.00219.

84. Blanchet FG, Cazelles K, Gravel D. 2020. Co-occurrence is not evidence of ecological interactions. Ecol Lett 23:1050-1063. https://doi.org/10.1111/ele.13525.

85. Fuhrman JA. 2009. Microbial community structure and its functional implications. Nature 459:193-199. https://doi.org/10.1038/nature08058.

86. Liu LH, Zhang JY, Tang GX, Huang YH, Xie XQ, Geng J, Lü HX, Li H, Li YW, Mo CH, Zhao HM, Cai QY. 2023. Endophytic Phthalate-degrading *Bacillus subtilis* N-1-gfp colonizing in soil-crop system shifted indigenous bacterial community to remove di-n-butyl phthalate. J Hazard Mater 449:130993. https://doi.org/10.1016/j.jhazmat.2023.130993.

87. Bai J, Ding Z, Su R, Wang M, Cheng M, Xie D, Guo X. 2022. Storage Temperature Is More Effective Than Lactic Acid Bacteria Inoculations in Manipulating Fermentation and Bacterial Community Diversity, Co-Occurrence and Functionality of the Whole-Plant Corn Silage. Microbiol Spectr 10:e0010122. https://doi.org/10.1128/spectrum.00101-22.

88. Fukami T. 2015. Historical contingency in community assembly: integrating niches, species pools, and priority effects. Annu Rev Ecol Evol Syst 46:1-23. https://doi.org/10.1146/annurev-ecolsys-110411-160340.

89. Maignien L, DeForce EA, Chafee ME, Eren AM, Simmons SL. 2014. Ecological succession and stochastic variation in the assembly of *Arabidopsis thaliana* phyllosphere communities. mBio 5:e00682-13. https://doi.org/10.1128/mBio.00682-13.

90. Carlström CI, Field CM, Bortfeld-Miller M, Müller B, Sunagawa S, Vorholt JA. 2019. Synthetic microbiota reveal priority effects and keystone strains in the Arabidopsis phyllosphere. Nat Ecol Evol 3:1445-1454. https://doi.org/10.1038/s41559-019-0994-z.

91. Wippel K, Tao K, Niu Y, Zgadzaj R, Kiel N, Guan R, Dahms E, Zhang P, Jensen DB, Logemann E, Radutoiu S, Schulze-Lefert P, Garrido-Oter R. 2021. Host preference and invasiveness of commensal bacteria in the Lotus and Arabidopsis root microbiota. Nat Microbiol 6:1150-1162. https://doi.org/10.1038/s41564-021-00941-9.

92. Gu Y, Banerjee S, Dini-Andreote F, Xu Y, Shen Q, Jousset A, Wei Z. 2022. Small changes in rhizosphere microbiome composition predict disease outcomes earlier than pathogen density variations. ISME J 16:2448-2456. https://doi.org/10.1038/s41396-022-01290-z.

93. Walsh CM, Becker-Uncapher I, Carlson M, Fierer N. 2021. Variable influences of soil and seed-associated bacterial communities on the assembly of seedling microbiomes. ISME J 15:2748-2762. https://doi.org/10.1038/s41396-021-00967-1.

94. Aneja MK, Sharma S, Munch JC, Schloter M. 2008. Importance of DNA quality in comparative soil microbial community structure analyses. Soil Biol Biochem 40:1390-1403. https://doi.org/10.1016/j.soilbio.2007.12.027.

95. Bolyen E, Rideout JR, Dillon MR, Bokulich NA, Abnet CC, Al-Ghalith GA, Alexander H, Alm EJ, Arumugam M, Asnicar F, Bai Y, Bisanz JE, Bittinger K, Brejnrod A, Brislawn CJ, Brown CT, Callahan BJ, Caraballo-Rodriguez AM, Chase J, Cope EK, Da Silva R, Diener C, Dorrestein PC, Douglas GM, Durall DM, Duvallet C, Edwardson CF, Ernst M, Estaki M, Fouquier J, Gauglitz JM, Gibbons SM, Gibson DL, Gonzalez A, Gorlick K, Guo J, Hillmann B, Holmes S, Holste H, Huttenhower C, Huttley GA, Janssen S, Jarmusch AK, Jiang L, Kaehler BD, Kang KB, Keefe CR, Keim P, Kelley ST, Knights D, et al. 2019. Reproducible, interactive, scalable and extensible microbiome data science using QIIME 2. Nat Biotechnol 37:852-857. https://www.ncbi.nlm.nih.gov/pubmed/31341288.

96. Edgar RC. 2010. Search and clustering orders of magnitude faster than BLAST. Bioinformatics 26:2460-2461. https://doi.org/10.1093/bioinformatics/btq461.

97. Rognes T, Flouri T, Nichols B, Quince C, Mahé F. 2016. VSEARCH: a versatile open source tool for metagenomics. PeerJ 4:e2584. https://doi.org/10.7717/peerj.2584.

98. Edgar RC. 2016. UNOISE2: improved error-correction for Illumina 16S and ITS amplicon sequencing. BioRxiv doi:10.1101/081257:081257. https://doi.org/10.1101/081257.

99. Edgar RC. 2016. SINTAX: a simple non-Bayesian taxonomy classifier for 16S and ITS sequences. BioRxiv doi:10.1101/074161:074161. https://doi.org/10.1101/074161.

100. Oksanen J, Kindt R, Legendre P, O’Hara B, Wagner H. 2007. The vegan package. Community ecology package 10:631-637. https://cran.r-project.org/.

101. Ginestet C. 2011. ggplot2: Elegant Graphics for Data Analysis. J R Stat Soc Ser A Stat Soc 174:245-246. https://doi.org/10.1111/j.1467-985X.2010.00676_9.x

102. Love MI, Huber W, Anders S. 2014. Moderated estimation of fold change and dispersion for RNA-seq data with DESeq2. Genome Biol 15:550. https://doi.org/10.1186/s13059-014-0550-8.

103. Bastian M, Heymann S, Jacomy M. 2009. Gephi: an open source software for exploring and manipulating networks. Proceedings of the Third International Conference on Weblogs and Social Media 3:361-362. https://doi.org/10.13140/2.1.1341.1520.

104. Louca S, Parfrey LW, Doebeli M. 2016. Decoupling function and taxonomy in the global ocean microbiome. Science 353:1272-1277. https://doi.org/10.1126/science.aaf4507.

105. Price MN, Dehal PS, Arkin AP. 2009. FastTree: computing large minimum evolution trees with profiles instead of a distance matrix. Mol Biol Evol 26:1641-1650. https://doi.org/10.1093/molbev/msp077.

106. Price MN, Dehal PS, Arkin AP. 2010. FastTree 2-approximately maximum-likelihood trees for large alignments. PLoS One 5:e9490. https://doi.org/10.1371/journal.pone.0009490.

**Figure Legends**

**Fig. 1. Taxonomic composition of the bacterial communities.** (a) The relative abundances of the most abundant (sub)phylum level in each compartment between Xs and CK. b) Comparative analysis of relative abundance of Gammaproteobacteria in Xs and CK (paired *t*-test). “RCKC/ RXsC”, root endophytic microbes CK or Xs at maturity stage; “HCKM/ HXsM”, rhizosphere microbes CK or Xs at seedling stage; “RCKF/RXsF”, root endophytic microbes CK or Xs at tillering stage; “SCKF/SXsF”, stem endophytic microbes CK or Xs at tillering stage. The statistical analyses were performed using a two-sided T-test. *P* values are indicated by *, * represents p < 0.05, ** represents p < 0.01.

**Fig. 2. Taxonomic *α*- and *β*-diversity estimates.** (a) Shannon indices of bacterial communities in bulk soil, rhizosphere, root endosphere, stem endosphere, and seed endosphere. Horizontal lines within boxes denote medians. Tops and bottoms of boxes denote the 75th and 25th percentiles, respectively. Upper and lower whiskers extend to data no more than 1.5× the interquartile range from the upper edge and lower edge of the box, respectively. Different letters indicate significant differences among compartments (*P*< 0.05), based on Kruskal-Wallis one-way test. b) PCoA plot depicting the *β*-diversity patterns of bacterial communities across different compartments based on Bray-Curtis dissimilarity. C) *α*-Diversity CK in each compartment at the seedling stage, tillering stage, booting stage, and maturity stage compared with Xs based on the Wilcoxon rank sum test. Shannon and richness index represent Mean ± SD according to group, “Ns” represents not significantly difference. D) PCoAs of microbial community composition in individual compartments at each development stages between Xs and CK based on the Bray-Curtis dissimilarity. Significant difference between treatments with and without *Xanthomonas* inoculation based on PERMANOVA and adonis function.

**Fig. 3. Effect of early inoculation on network complexity and positive-related connections.** (a) Bacterial co-occurrence networks in root and stem endophytic compartments (90 samples). b) Bacterial co-occurrence network characteristics in each compartment niche. The size of each node represents the size of average degree of the microbe. Link color indicates the type of correlation: blue, positive correlation; red, negative correlation.

**Fig. 4. Enrichment of genera inhabiting different plant microhabitats.** (a) The volcano plot illustrates the enrichment and depletion of microbial communities in each compartment of Xs compared to CK at each growth stage using the deseq2 package. Genera were considered enriched if they showed a log2-fold change greater than 2 and an adjusted *P*-value less than 0.05. Each point represents a genus. Each red point represents enriched genus, and blue points represent depleted genera. b) Venn plot illustrating the five genera that were significantly co-enriched at the seedling stage compared with CK in root endosphere, stem endosphere, and rhizosphere. c) The distribution pattern of actual abundance of the four co-enriched genera in each compartment at the developmental stages. The letter "S" in x-axis represents " seedling stage "; “T”, tillering stage; “B”, booting stage; “M”, maturity stage.

**Fig. 5. The microbial community assembly processes.** (a) The percentage of turnover in the rhizosphere, root and stem microbial communities, governed by “selection”, “dispersal” and “drift” processes. The position along the y-axis represents the process importance (“selection” is a combination of homogeneous selection and heterogeneous selection; “dispersal” is a combination of homogenizing dispersal and dispersal limitation; “drift” represents drift and others), and the x-axis represents the growth stages: 1, seedling stage; 2, tillering stage; 3, booting stage; 4, maturity stage. b) The community assembly importance in the rhizosphere, root, stem and seed microbial communities governed by homogeneous selection, heterogeneous selection, dispersal limitation, homogenizing dispersal and drift processes.

**Fig. 6. A conceptual model of the impact of early inoculation on microbiomes assembly and ecological processes over the developmental stages in the rice.**
